# The integrity of the nucleus of the lateral olfactory tract is essential for the normal functioning of the olfactory system

**DOI:** 10.1007/s00429-017-1422-2

**Published:** 2017-04-19

**Authors:** Ricardo P. Vaz, Armando Cardoso, Susana I. Sá, Pedro A. Pereira, M. Dulce Madeira

**Affiliations:** 1 Unit of Anatomy, Department of Biomedicine, Faculty of Medicine, University of Porto, Alameda Professor Hernâni Monteiro, 4200-319 Porto, Portugal; 2 Otorhinolaryngology Department, Centro Hospitalar S. João, EPE, Alameda Professor Hernâni Monteiro, 4200-319 Porto, Portugal; 3 Center for Health Technology and Services Research (CINTESIS), Rua Dr. Plácido da Costa, 4200-450 Porto, Portugal

**Keywords:** Aggression, Anosmia, Attractive and avoidance behaviors, Cortical pallial amygdala, Olfactory cortex, Sexual behavior

## Abstract

The nucleus of the lateral olfactory tract (nLOT) is a relatively small component of the cortical pallial amygdala, with peculiar neurogenic, neurochemical and connectivity patterns. Although it has been suggested that it might be involved in non-pheromonal olfactory-guided behaviors, particularly feeding, the functional implications of the nLOT have never been investigated. In view of this fact, we have tackled this subject by performing a series of behavioral tests and by quantifying biological and biochemical parameters in sexually naïve adult male rats that were submitted to bilateral excitotoxic lesions of the nLOT. nLOT-lesioned rats had severe olfactory deficits with inability to detect and discriminate between odors. Additionally, they did not display innate behavioral responses to biologically relevant chemosignals. Specifically, nLOT-lesioned rats did not show avoidance towards predator odors or aggressive behaviors towards intruders, and had severely impaired sexual behavior. In fact, nLOT lesions abolished preference for odors of receptive females, reduced chemoinvestigatory behavior and eliminated mounting behavior. nLOT-lesioned rats had normal circulating levels of testosterone, did not display anxiety- or depressive-like behaviors, and had unimpaired cognitive functions and fear acquisition and memory. Altogether, our results suggest that the nLOT integrity is required for the normal functioning of the olfactory system.

## Introduction

The nucleus of the lateral olfactory tract (nLOT) is a small three-layered structure, with a volume of 0.24 mm^3^ and 19,000 neurons (Vaz et al. 2016). It is located in the ventral surface of the brain interposed between the anterior amygdaloid area and the anterior medial amygdala (Ennis et al. 2015), and is related laterally with the anterior cortical amygdaloid nucleus (de Olmos et al. 2004). Because it has a cortical structure and receives a direct projection from the main olfactory bulb, some authors have regarded it as a component of the olfactory cortex (Price 1973; Swanson and Petrovich 1998). Due to its topographical relationships, other authors have considered it as one component of the olfactory amygdala, which also includes the anterior amygdaloid area, the anterior and the posterior cortical amygdaloid nuclei and the amygdalo-piriform transition area (reviewed in de Olmos et al. 2004). According to contemporary classification of amygdala nuclei (reviewed in Olucha-Bordonau et al. 2015), which is based on the conjoint analysis of morphological and neurochemical data, connectivity and pattern of gene expression during brain development, the nLOT has been included in the cortical pallial amygdala. It shares this classification with the anterior, the posteromedial and the posterolateral cortical nuclei, the bed nucleus of the accessory olfactory tract, and the cortico-amygdala and amygdalo-piriform transition areas. However, the nLOT does not fit completely within this group due to its atypical features of development. First, it develops later than the remaining components of the amygdala (Müller and O’Rahilly 2006). Second, it has a mixed origin with its layer 1 originating from the ventral pallium and layers 2 and 3 possibly from the dorsal pallium (Remedios et al. 2007; Subramanian et al. 2009) or the lateral pallium (Puelles et al. 2000; Gorski et al. 2002; Medina et al. 2004), whereas the remaining components of the cortical pallial amygdala are derivatives either from the lateral or the ventral pallium (Medina et al. 2004; Olucha-Bordonau et al. 2015).

Along with its peculiar neurogenic pattern, the nLOT also stands up as a unique region within the group of the cortical pallial nuclei due to some particular aspects of its morphology, neurochemistry and connections. It displays a distinctive three-layered organization (Price 1973; McDonald 1983; Millhouse and Uemura-Sumi 1985; Vaz et al. 2016) and its layer 2 neurons, which are relatively large and represent over 80% of the total neuronal population of the nLOT (Vaz et al. 2016), express a type of vesicular glutamate transporter (type 2) that is considerably less abundant, or even non-existent, in other areas of the pallial amygdala (Hur and Zaborszky 2005). It has a much higher density of heavy metals (Friedman and Price 1984) and stains more intensely for cholinergic markers (Millhouse and Uemura-Sumi 1985; Vaz et al. 2016) than the surrounding structures. Unlike other components of the olfactory amygdala (Kevetter and Winans 1981; Swanson and Petrovich 1998; McDonald 2003; Gutiérrez-Castellanos et al. 2014), it does not send direct projections to the extended amygdala or the hypothalamus (Santiago and Shammah-Lagnado 2004). Instead, it is bi-directionally connected with the olfactory bulb and the piriform cortex and strongly innervates the basolateral amygdala and ventral striatum, with some fibers being conveyed to the prefrontal and insular cortices (Price 1973; Luskin and Price 1983; McDonald 1991; Jolkkonen et al. 2001; Santiago and Shammah-Lagnado 2004). Due to these connections, the nLOT has been thought of as being involved in non-pheromonal olfactory-guided behaviors, especially feeding (Petrovich et al. 1996; Cardinal et al. 2002; Santiago and Shammah-Lagnado 2004). However, no study has so far attempted to examine in detail the functional significance of the nLOT.

To begin to address this issue, we conducted a battery of behavioral tests in adult male rats submitted to excitotoxic lesions of the nLOT and compared the data with those obtained in sham-lesioned and control rats. Because, in addition to receiving afferents from the main olfactory bulb, the nLOT also receives direct and limited indirect inputs—via the posteromedial cortical amygdaloid nucleus—from the accessory olfactory bulb (Pro-Sistiaga et al. 2007; Gutiérrez-Castellanos et al. 2014), we have assessed its involvement in olfaction, innate reproductive (e.g., mating) and defensive (e.g., predator avoidance and aggression) behaviors, and also in sensorimotor, anxiety- and depression-like behaviors, fear conditioning, learning and memory.

## Materials and methods

### Animals

Sexually naïve male Wistar rats, derived from the Institute for Molecular and Cell Biology (Porto, Portugal), were used. After acclimation to laboratory conditions for at least 1 week, rats were single-housed and maintained in standard environmental conditions (12-h light/dark cycles with lights on at 7:00 a.m., ambient temperature of 21 ± 1 °C, 45 ± 5% relative humidity) with ad libitum access to food and water, unless specifically noted. All experiments were carried out in accordance with the guidelines of the European Communities Council Directives of 22 September 2010 (2010/63/EU) and Portuguese Act n^o^113/13, and approved by ORBEA, the internal committee of the Faculty of Medicine, University of Porto (Portugal). Body weights were determined weekly before the experiments, on the day of surgery and once per week thereafter. To determine the average 24-h baseline food and water intake, the amount of food and water ingested was measured daily. At 10 weeks of age, rats were randomly assigned to one of three groups: control, nLOT-lesioned and sham-lesioned. nLOT- and sham-lesioned rats were allowed 10 days to recover prior to the start of behavioral testing.

### Surgical procedures and stereotaxic injections

Rats were anesthetized by sequentially injecting, at intervals of 10 min, solutions of promethazine (10 mg/kg, s.c.; Laboratórios Vitória, Amadora, Portugal), followed by xylazine (2.6 mg/kg, i.m.; Sigma-Aldrich Company Ltd., Madrid, Spain), and finally, ketamine (50 mg/kg, i.m.; Merial Portuguesa, Rio de Mouro, Portugal), and placed on a stereotaxic apparatus with bregma and lambda in the same horizontal plane. nLOT lesions were made by bilateral infusion of 0.24 µL of quinolinic acid (Sigma-Aldrich). The acid was dissolved in 0.1 M of phosphate buffered saline (PBS) to a concentration of 180 nmol/µL (pH 7.1). The infusion of the toxin was done with a 1-µL Hamilton syringe (7001 N; Hamilton Bonaduz AG, Bonaduz, Switzerland). After a midline skin incision, holes were drilled bilaterally on the skull 1.2 mm posterior and ±3.2 mm laterally to the bregma (Paxinos and Watson 1998). The syringe was lowered into the brain until the depth of 9.2 mm from the bregma. The toxin was injected gradually (0.04 µL every 1.5 min) until the total amount was delivered. The needle was left in place for an additional 10 min, and then slowly withdrawn. The incisions on the skin were closed with surgical stitches and treated with local antiseptic. After surgery, rats were maintained in a warm place until recovery from anesthesia. Postoperative care consisted of subcutaneous injections of 0.9% physiological saline (2 mL) to prevent dehydration and weight loss. Sham lesions were similarly made by lowering the infusion needle at the same coordinates without infusing quinolinic acid.

### Behavioral studies

**Fig. 1:**
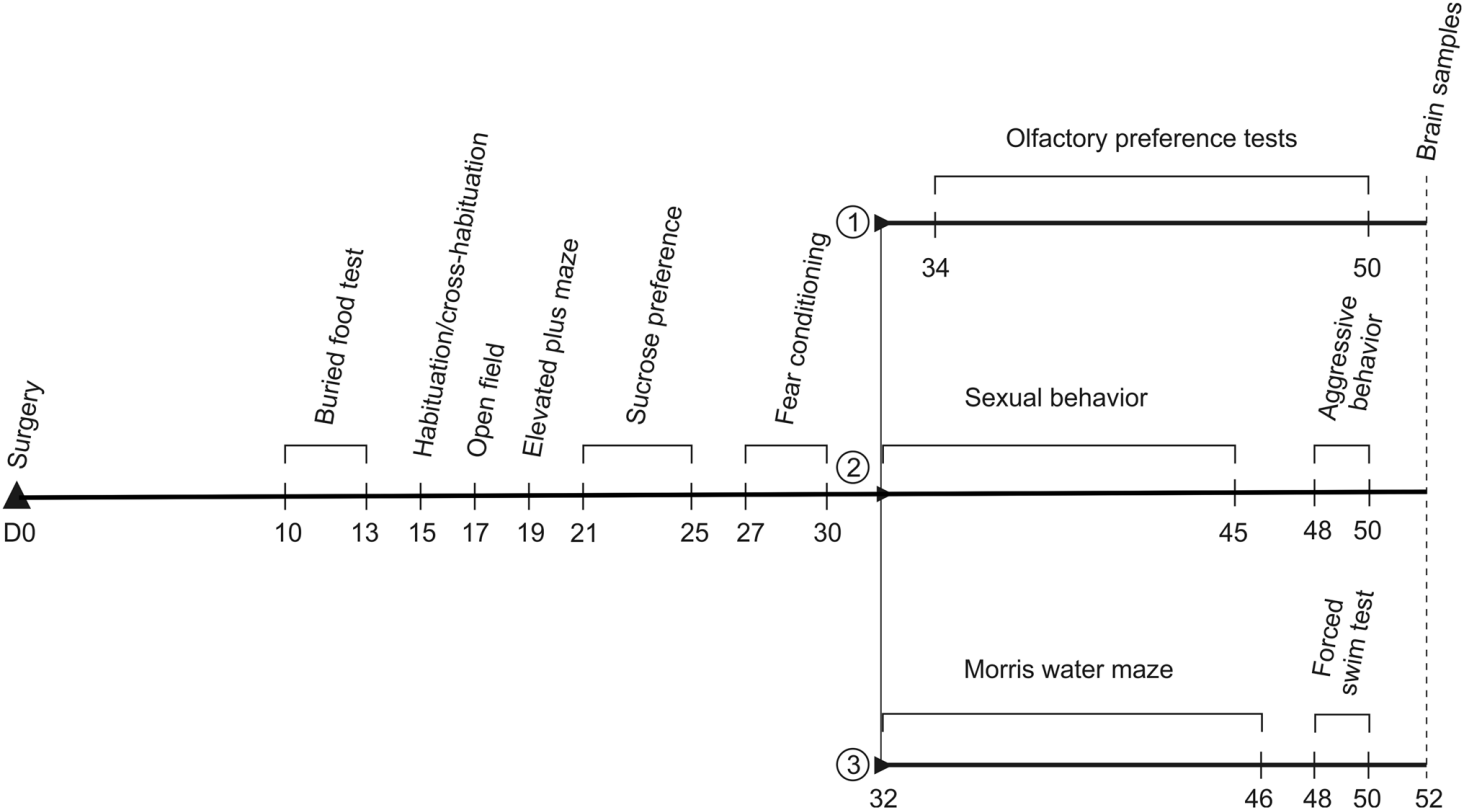
Sequence and experimental timeline of the behavioral tests. The day of surgery was used as day 0 (D0) of the experiments. Control rats were age-matched. Tests were all done with 1-day inter-test intervals. Tests done until day 30 post-surgery included all rats (*n* = 30/group). Thereafter, one third of the rats in each group (*n* = 10/group) were submitted either to (*1*) olfactory preference tests, (*2*) sexual behavior followed by aggression, or (*3*) Morris water maze followed by forced swim test

All behavioral experiments were conducted during the standard light phase, starting at 2:00 p.m., except for sexual behavioral and aggression testing that was started 1 h after the beginning of the dark phase. For control (*n* = 30), sham-lesioned (*n* = 30) and nLOT-lesioned (*n* = 30) rats, tests were performed in the following order, with 1-day inter-test intervals: buried food test, olfactory habituation/cross-habituation test, open-field, elevated plus-maze, sucrose preference and fear conditioning. At the end of these tests, one third of the rats in each group (*n* = 10/group) were submitted either to (1) olfactory preference tests, (2) sexual behavior followed by aggression, or (3) Morris water maze followed by forced swim test (Fig. 1).

#### Buried food test

To assess the ability of the rats to smell volatile odors and their tendency to use olfactory cues for foraging, the buried food test (Yang and Crawley 2009) was performed. For odor familiarization, a highly palatable cookie was placed in the test chamber during two consecutive days before the test, and confirmed that it was consumed by the rat. The chamber consisted of a clean standard plastic cage (44 × 34 × 20 cm) with a 5 cm layer of new bedding. After 18 h of food deprivation, the rat was placed in the chamber for 10 min to acclimate. Then, the rat was removed from the cage and a cookie was buried in the bedding, approximately 2 cm beneath the surface, at a random location. The bedding surface was smoothed out and the rat was re-introduced into the cage. The time spent to locate the buried cookie was recorded. The maximum test time allowed was 900 s. Two hours later, a surface cookie test was performed. This test was set up in the same way as the buried food test, but the cookie was placed on the surface of the bedding instead of being buried.

#### Olfactory habituation/cross-habituation test

To assess the ability to detect and discriminate between a familiar and a novel odor, the test was performed as previously described (Yang and Crawley 2009). Prior to testing, the rat was allowed to acclimate for 10 min to a clean cage (38 × 24 × 20 cm), in which a plastic tube applicator (10 × 0.5 cm), containing a rectangular filter paper inside (3 × 1 cm), was inserted through the water bottle hole. We used this kind of applicator to avoid direct contact with the odor stimulus. The test consisted of sequential presentations of water and four different odors in three consecutive trials of 2 min, with 1-min inter-trial intervals. The sequence used was water, two nonsocial odors (100 µL lemon extract, 1:10 dilution in distilled water, and 100 µL strawberry extract, 1:10 dilution in distilled water) and two different social male odors (100 µL of social odor solution). Each social odor was obtained by swabbing, with a cotton tip, the bedding of two different cages with male rats and diluting it in a glass vial with water and with 100 µL of urine collected from each of the males in those cages. The cumulative time spent sniffing the odorant in each presentation was quantified with a stopwatch within a 2-min time period.

#### Open-field test

To assess general exploratory locomotion and anxiety-like behaviors, we used an open-field apparatus that consisted of a white acrylic arena (100 × 100 × 40 cm). The rat was placed in a corner of the apparatus and tested during 5-min sessions. Distances travelled in the outer zone of the open-field, defined as 20 cm from any wall, and in its inner zone, defined as the 60 × 60 cm square in the center of the arena, were measured using a computerized video-tracking system (EthoVision XT 8.5, Noldus, The Netherlands). At the end of each session, the number of fecal boli deposited was counted and recorded, and the urine deposited was collected using a filter paper. The difference between the weight (in g) of the paper before and after collecting the urine was considered as a measure of the amount of urine deposited during the session. The floor of the apparatus was then thoroughly cleaned and dried.

#### Elevated plus-maze

To further evaluate general exploratory and anxiety-like behaviors, an elevated plus-maze apparatus consisting of a black acrylic cross with two opposite open and two opposite closed arms (50 × 12 cm) joined by a common central square (12 × 12 cm) was used. The closed arms were enclosed by 50-cm high walls. The test rat was placed on the central square facing one of the closed arms and allowed to explore the apparatus for 5 min. The behavior of the rat was recorded and analyzed using a computerized video-tracking system (EthoVision XT 8.5, Noldus). The percentages of time spent and the distances travelled by rats in the open arms, in the closed arms and in the central square were computed. At the end of each session, the number of fecal boli and the amount of urine were recorded. The apparatus was then thoroughly cleaned and dried.

#### Sucrose preference test

To assess possible anhedonia (decreased sensitivity to reward), generally associated to depression, rats were subjected to the sucrose preference test. During four consecutive days, rats were given the choice to drink from two bottles placed side-by-side, one containing water and the other a 2% of sucrose (Sigma-Aldrich) solution. One day before the test, rats were acclimated to the two-bottle configuration and to sucrose taste. The total amount of liquid consumed by rats was measured every day and fresh solutions were prepared. To avoid possible effects of bottle side bias, the position of the bottles was switched every 12 h. Sucrose preference was calculated as a percentage of the volume of sucrose solution intake relative to the total volume of fluid consumption and averaged over the 4-day testing period. The increase in the cumulative intake of water and sucrose solution during the 4-day test period was calculated by relation to the fluid intake measured under baseline conditions.

#### Fear conditioning

To assess the acquisition, consolidation and expression of fear conditioned responses, all rats were given a single session of fear conditioning, as previously described (Cardoso et al. 2009). The conditioning chamber (San Diego Instruments, USA) consisted of a clear Plexiglas box (26 × 26 × 18 cm) equipped with a metal grid floor connected to a stimulus generator (Hugo-Sachs Elektronik, Germany) and a buzzer. The grid floor was made of stainless steel bars (0.6 cm diameter) spaced 1.4 cm apart. An olfactory cue was added by placing 1% of acetic acid solution on the metal tray beneath the grid floor. On day 1, rats were allowed to explore the test chamber for 3 min. During the next 3-min period, they received 5 tone-footshock conditioning trials, with 30-s intervals. In each conditioning trial, rats were exposed to the conditioned stimulus (2.8 kHz, 80 dB tone lasting 10 s) co-terminated with the unconditioned stimulus (0.8 mA of a continuous 1-s footshock). Thirty seconds after the last trial, rats were removed from the apparatus and the grid floor was cleaned with 1% acetic acid. Twenty-four hours later, half of the rats in each group were tested for contextual fear memory and the other half for cued fear memory. On the next day, rats already tested for contextual fear memory were tested for cue fear memory and vice versa. To test for contextual fear memory, rats were placed in the training chamber and were monitored for 6 min. To test for cued fear memory, rats were placed in a novel chamber, where they were left undisturbed for 3 min; then, during the next 3 min, they were exposed five times to the conditioned stimulus. In this test, the novel chamber was located in a novel behavioral room and was composed of black Plexiglas, except the top that was translucent and the floor that consisted of a piece of a black carpet and was scented with lemon instead of acetic acid. Training and all testing trials were recorded with a video camera for subsequent analysis. Freezing (defined as the absence of all movement other than that required for breathing and associated with a crouching posture) was scored if the rat remained inactive for at least 3 s. The percentage of accumulated time spent freezing was calculated.

#### Olfactory preference tests

Rats were allowed to habituate to the testing room and to the open-field arena during 10 min for 2 days prior to testing. Then, they were submitted, first, to a series of single odorant tests, and lastly, to the triple odorant test. Rats were exposed to one stimulus per day. They were naïve to each odor (as the odors tested were different from those used in the habituation/cross-habituation test) and were tested only once for each odor (at each concentration).

In the single odor test, the open-field arena contained Petri dishes in each corner, with only one Petri dish containing the odor stimuli. In the two habituation trials, the conditions were the same of the experimental test, except that no odor was used. Exposure to the odor was done by placing a 3.5-cm covered Petri dish containing a piece of filter paper (2 × 2 cm) impregnated with the odor stimuli in one corner of the open-field apparatus. As done by other authors (Dewan et al. 2013), the top of Petri dish was perforated to allow odorants to escape and to prevent direct nasal contact. One Petri dish containing a filter paper without any odor was placed in each of the other corners of the open-field arena. Because animals were tested in the same open-field arena to all odors, each of these odors was introduced in a pseudo-random corner of the arena to avoid any corner bias. Then, the test rat was placed in the center of the arena and allowed to freely explore the apparatus during 15 min. Sessions were video-recorded and later analyzed. The time spent by the rat actively investigating the corner where the odor was delivered was recorded and compared with the time rats spent investigating water. The odorants tested were 2-phenylethanol (2PE, 50% in water), 2-phenylethylamine (PEA, 50% in water), isopentylamine (IPA, 10% in water), 2,5-dihydro-2,4,5-trimethylthiazoline (TMT, 2% in water) and cat fur odor (CFO). With the goal of testing whether odor concentration would affect odor preference, we have also performed the test using concentrations that were twice higher for 2PE (100%), PEA (100%), IPA (20%) and five times larger for TMT (10%). Chemicals were all purchased from Sigma-Aldrich, except TMT, which was purchased from SRQBio (USA). Cat fur was collected from a domestic adult male cat.

For the triple odor exposure, each rat was simultaneously presented with urine from group-housed testes-intact male rats, females in behavioral estrus and diestrus female rats. Urine was collected using metabolic cages. Urine from each group was pooled, aliquoted and stored at −80 °C until use. All the procedures were identical to the single odor test, except that in this test three odors and water were presented simultaneously, one in each corner. After placing the water and the three odor stimuli in the corners of the open-field cage, the test rat was placed in the center of the arena and allowed to freely explore for 15 min. Sessions were video-recorded and later analyzed. The time spent actively investigating each corner of the open-field was recorded.

#### Sexual behavior

Rats were observed in pre-copulatory behaviors and mounting in three assays with 4-days inter-trial intervals. On each trial, the test rat was placed into the cage and allowed to acclimate for 10 min. A sexually experienced receptive female rat was then introduced into the cage. Females were brought into behavioral estrus by subcutaneous injections of 10 μg of estradiol benzoate 52 h before the test, followed by 500 μg of progesterone 4–6 h before testing. Sexual behavior was recorded with a video camera during a 10-min period. The latency to the first anogenital exploration and to the first mounting/intromission was recorded from the time the female entered the cage. The cumulative duration of anogenital exploration, sniffing and rearing, and female pursuit was also recorded.

#### Aggressive behavior

Male aggression was assessed by the resident/intruder assay (Leypold et al. 2002). Rats in all groups were single-housed since the beginning of the study. The test rat was maintained in its home cage without changing of the bedding for the previous 4 days. A group-housed, sexually and aggressively inexperienced and unfamiliar adult rat, lighter than the test rat, was introduced into the home cage of the test rat. The tests were video-recorded over 15 min, and later analyzed to determine the duration of offensive behaviors (attack, offensive upright, lateral threat, keep down) and defensive behaviors (move away, submissive posture, defensive upright).

#### Morris water maze

To assess spatial learning and memory, rats were tested in a black circular pool (180 cm diameter; 50 cm deep) filled with water at room temperature (21 ± 1 °C) that was located in a corner of a room containing extra-maze cues. The pool was virtually divided into four equal-size quadrants. A black escape platform (10 cm in diameter) was placed in the center of one of the quadrants, 2 cm below the water surface. Swim paths were recorded by a computerized video-tracking system (EthoVision XT 8.5, Noldus). In the place learning task, rats were trained to find the submerged escape platform and to climb on it. For acquisition, rats were given two trials per day for 14 consecutive days, as follows. The test rat was placed in the water facing the pool wall at one of four starting points, which were used in a pseudo-random order so that each position would be used just once in each block of four trials. When rats did not find the escape platform within 60 s, the experimenter guided them to the platform where they were allowed to remain for 15 s. After the first daily trial, rats were placed in a clean cage for 30 s before the beginning of the next trial. The platform location was not changed during the acquisition period. The swim path length in each trial was measured. One day after the end of the acquisition period, rats were submitted to a single 60-s probe trial, in which the platform was removed from the pool. The number of times the rats swam through the zone where the platform had been located (platform crossings) and the time they spent swimming on the target and opposite quadrants were recorded. Starting one day later, all rats were tested during a 2-day period on the visible platform task to evaluate their sensorimotor abilities. In this task, rats were given 1 block of four trials per day separated by 30-s inter-trial intervals. The platform, painted in white, was exposed 3 cm above the water surface and its position was different in each trial. The distances swum to locate the platform were recorded and averaged across eight trials.

#### Forced swim test

To assess the depression state of animals, the forced swim test was done essentially as previously described (Porsolt et al. 1978). The apparatus used consisted of a transparent glass cylinder (25 cm diameter, 50 cm height) filled with tap water (23–25 °C) up to 30 cm from the bottom. On the first trial, the test rat was introduced into the apparatus and forced to swim during 15 min. On the day after, a second trial was performed during 5 min, and the behavior was recorded using a digital video camera. The total duration of immobility during the 5-min long testing period was recorded, and the percentage of accumulated time that rats were immobilized was calculated. Immobility was defined as the absence of movements beyond those required for keeping the head and nose above the water surface.

### Brain tissue collection and histology

Following behavioral testing, rats were anesthetized with sevoflurane (SevoFlo, Abbott Laboratories Ltd, Maidenhead, UK) and blood samples were collected directly from the heart for hormone measurements. Then, rats were killed by transcardiac perfusion of 150 mL of 0.1 M phosphate buffer (PB), pH 7.6, for vascular rinse, followed by 250 mL of a fixative solution containing 4% paraformaldehyde in PB. The gonadal, perirenal and retroperitoneal fat depots were isolated, collected and weighed. The brains were removed from the skulls, immersed for 1 h in the same fixative, and maintained overnight in a solution of 10% sucrose in PB, at 4 °C. After removal of the frontal and occipital poles and separation of the right and left hemispheres, the remaining blocks of tissue were coronally sectioned at 40 µm on a vibratome throughout the rostrocaudal extent of the nLOT. Sections were alternately sampled, mounted on gelatin-coated slides, air-dried, either Giemsa- or Cresyl Violet (Nissl)-stained (Merck, Darmstadt, Germany), dehydrated and coverslipped with Histomount (National Diagnostics, USA). Lesion placements were verified by microscopic examination, and drawn onto plates adapted from the atlas of Paxinos and Watson (1998). To assess the percent destruction of the nLOT, the volume of the nLOT cell layers and the volume of the lesions were estimated by point counting techniques (Gundersen and Jensen 1987; Madeira et al. 1997).

### Imaging

**Fig. 2:**
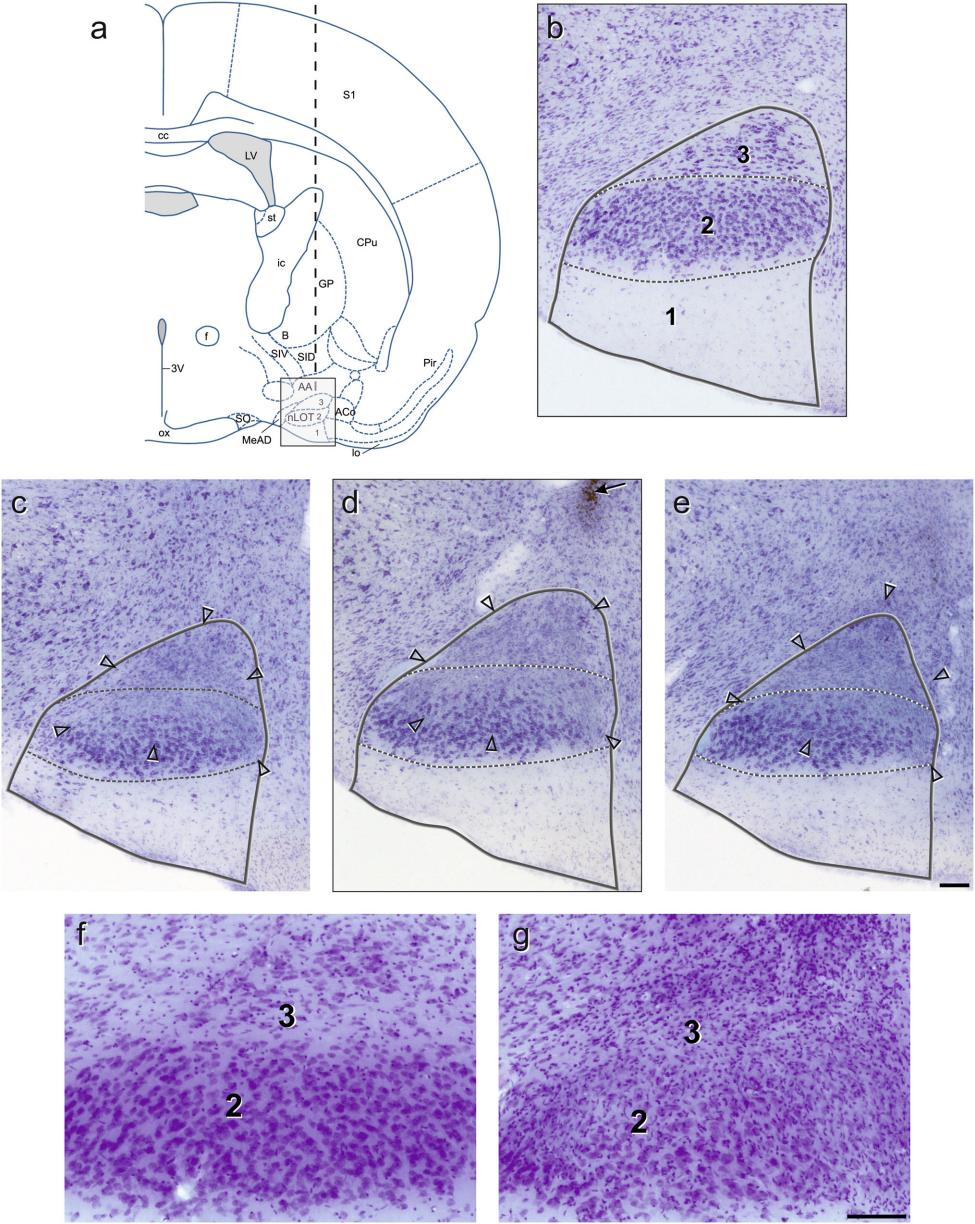
**a** Schematic drawing of a coronal section of the rat brain through the nLOT (adapted from Paxinos and Watson 1998). The *thick dashed vertical line* indicates the trajectory of the needle. (**b–e**) Digital photomicrographs of Giemsa-stained coronal sections of the nLOT of a sham-lesioned rat (**b**) and a nLOT-lesioned rat (**c–e**). The *light-gray box* drawn in **a** delineates approximately the area where the photomicrographs shown in **b** and **d** were taken. The **c** and **e** sections are located 160 μm rostral and caudal, respectively, to the **d** section. In **b–e**, the nLOT is outlined by a *continuous line* and the borders between the adjacent layers are indicated by *dashed lines*. The *open arrowheads* in **c–e** demarcate the periphery of the lesion and the *arrow* in **d** indicates the injection track. (**f–g**) Photomicrographs of Nissl-stained coronal sections of the nLOT layers* 2* and* 3* of a sham-lesioned rat (**f**) and a nLOT-lesioned rat (**g**) taken at a higher magnification than those shown in **b–e** to demonstrate that, by comparison with section of the sham-lesioned rat (**f**), there are numerous reactive glial cells in the nLOT-lesioned rat (**g**). *3V* 3rd ventricle, *AA* anterior amygdaloid area, *ACo* anterior cortical amygdaloid nucleus, *B* basal nucleus (Meynert), *cc* corpus callosum, *CPu* caudate putamen (striatum), *f* fornix, *ic* internal capsule, *GP* globus pallidus, *lo* lateral olfactory tract, *LV* lateral ventricle, *MeAD* medial amygdaloid nucleus, anterodorsal part, *nLOT* nucleus of the lateral olfactory tract (layers* 1*,* 2* and* 3*), *ox* optic chiasm, *Pir* piriform cortex, *S1* primary somatosensory cortex, *SID* substantia innominata, dorsal part, *SIV* substantia innominata, ventral part, *SO* supraoptic nucleus, *st* stria terminalis. *Scale bars* 100 μm

**Fig. 3:**
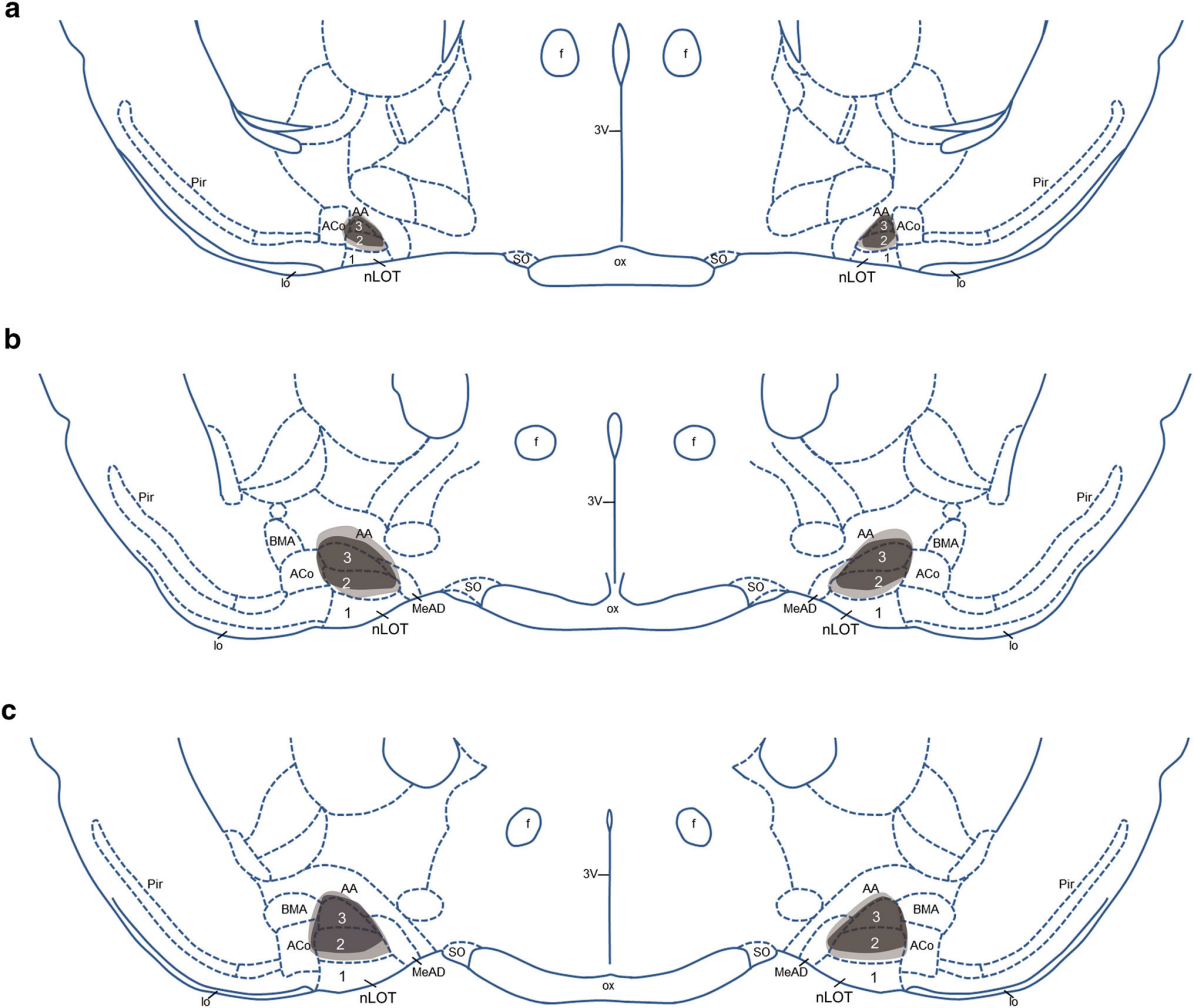
Rostrocaudal sequence **a–c** of schematic drawings of coronal sections of the rat brain through the nLOT (adapted from Paxinos and Watson 1998). Shaded areas represent the maximal (*light gray*) and the minimal (*dark gray*) extent of the nLOT lesions that were observed in the rats included in the study. *3V* 3rd ventricle, *AA* anterior amygdaloid area, *ACo* anterior cortical amygdaloid nucleus, *BMA* basomedial amygdaloid nucleus, anterior part, *f* fornix, *lo* lateral olfactory tract, *MeAD* medial amygdaloid nucleus, anterodorsal part, *nLOT* nucleus of the lateral olfactory tract (layers* 1*,* 2* and* 3*), *ox* optic chiasm, *Pir* piriform cortex, *SO* supraoptic nucleus

Schematic presentations of the experimental design (Fig. 1), the coronal sections of the rat brain through the nLOT (Figs. 2a, 3) were made with Corel Draw X8 (version 18.1.0.661; Corel, Ottawa, CA, USA). The photomicrographs shown in Fig. 2b–g were captured by digital photography using a Axio Scope A1 microscope with an AxioCam MRc5 digital camera, the AxioVision software (version 4.8.1.0; Carl Zeiss, Göttingen, Germany) and the following objectives: 10x in Fig. 2b–e and 20× in Fig. 2f, g. Only minor adjustments of contrast and brightness were made using Adobe Photoshop 7.0 (Adobe Systems, Mountain View, CA, USA), without altering the appearance of the original materials. The plate photomontage and lettering were made with Corel Draw X8.

### Hormone measurements

The blood samples collected at the time of killing were centrifuged. The serum was collected and immediately frozen at −80 °C until assayed. Leptin was measured using a commercial ELISA kit (Rat Leptin ELISA Kit; ab100773). Sensitivity of the test was 30 pg/mL and the intra and inter-assay coefficient of variation was 5.75 and 8.98%, respectively. Testosterone was determined by enzyme-linked fluorescent assay using VIDAS Testosterone Kit and mini VIDAS analyzer (bioMerieux S.A., Marcy L’Etoile, France). According to the manufacturer, the assay has a measurement range of 0.1–13 ng/mL. The analyzer was cleaned, calibrated, and operated in accordance with the manufacturer’s instructions. For testosterone, all samples were tested in singlicate, analyzed in the same assay and the intra-assay coefficient of variation was 7.44%.

### Statistical analyses

All figures represent means with the error bars representing SEM. Statistical analyses were performed using GraphPad Prism version 7.02 for Windows (GraphPad Software, La Jolla, CA, USA). Repeated measures analysis of variance (ANOVA) was used to analyze data on body weights (independent variables: post-surgery time and treatment; dependent variable: body weight), relative body weight change (independent variables: post-surgery time and treatment; dependent variable: body weight change relative to pre-lesion body weight), relative food intake (independent variables: post-surgery time and treatment; dependent variable: relative food intake) and fear conditioning (independent variables: context, tone and treatment; dependent variable: time spent in freezing). Two-way ANOVA was used for analysis of data obtained in the buried food test (independent variables: cookie location, treatment; dependent variable: time spent to find the cookie), open-field (independent variables: zone and treatment; dependent variables: distance travelled, number of fecal boli and grams of urine) and elevated plus-maze (independent variables: zone and treatment; dependent variables: distance travelled, time spent, number of fecal boli and grams of urine). One-way ANOVA was used to statistically analyze body fat mass and hormone levels (independent variable: treatment; dependent variables: fat mass, leptin levels, testosterone levels), and data from the sucrose preference test (independent variable: treatment; dependent variables: sucrose preference and fluid intake increase), forced swim test (independent variable: treatment; dependent variable: time spent in immobility), sexual behavior (independent variable: treatment; dependent variables: latency to anogenital exploration, latency to first mount, cumulative time spent in anogenital exploration, time spent sniffing and rearing and time spent in female pursuit), aggressive behaviors (independent variable: treatment; dependent variable: time spent in offensive behaviors) and defensive behaviors (independent variable: treatment; dependent variable: time spent in defensive behaviors). Data on olfactory habituation/cross-habituation was analyzed using repeated measures ANOVA (independent variables: water, lemon, strawberry, social 1, social 2, treatment; dependent variable: time spent sniffing the odor), whereas the cumulative time spent by rats sniffing each odor over the three trials was analyzed using a one-way ANOVA. Data obtained in the single odor test applied to examine olfactory preference, compared to water, was analyzed using two-way ANOVA (independent variables: 2PE, PEA, IPA, CFO, TMT, treatment; dependent variable: time spent in odor corner), whereas the triple odor test was analyzed separately for each treatment group by one-way ANOVA (independent variables: water, non-receptive female, receptive female and male odors; dependent variable: time spent in the odor corners). Data obtained in the Morris water maze was analyzed using repeated measures ANOVA (independent variables: platform location, treatment; dependent variable: distance travelled to find the platform) as well as a two-way ANOVA (independent variables: platform and treatment; dependent variable: time spent in the target and opposite quadrants). Whenever appropriate, ANOVAs were followed by Tukey highest significant difference (HSD) post-hoc comparisons. Differences were considered to be statistically significant when *p* < 0.05.

## Results

### Lesion analysis

The injection tracks, the placement of each injection site, and the location and size of the lesions were subjected to rigorous histological examination through microscopic analysis of Giemsa- and Nissl-stained sections (Fig. 2) before analysis of the behavioral data. The needle track passed through the cerebral cortex, corpus callosum, caudate-putamen, internal capsule, globus pallidus, basal nucleus (Meynert), substantia innominata and the anterior amygdaloid area (Fig. 2a). Minor mechanical damage from the needle insertion was evident in both sham- and nLOT-lesioned rats. None of the sham-lesioned rats presented damage of the nLOT or neighboring structures (Fig. 2b, f). Lesions were classified as suitable if there was significant bilateral damage encompassing an area greater than 50% of the nLOT cell layers, and no noteworthy damage of the neighboring structures, namely the anterior amygdaloid area, the basomedial, anterior cortical and medial amygdaloid nuclei, the lateral olfactory tract, the piriform cortex or the hypothalamus. Rats that did not fulfill these criteria, either unilaterally or bilaterally, were not included in the study, i.e., rats in which lesions could not be identified accurately (*n* = 3) and rats that only had unilateral lesions (*n* = 2) or presented significant bilateral damage of neighboring structures (*n* = 3). In all nLOT-lesioned rats included in the study there was massive neuronal loss and gliosis in layer 3 with variable, but frequently prominent damage of layer 2 (Fig. 2c–e, g). Taking into account the size of the lesions in coronal sections and their extension along the rostrocaudal length of the nLOT, the lesions of the rats included in the study encompassed, on average, 65% of the nLOT cell layers. Representative examples of nLOT lesions are shown, in a rostrocaudal sequence, in Fig. 2c–e. The maximal and the minimal extent of the lesions are depicted, also in a rostrocaudal sequence, in Fig. 3.

### Body weight and composition

**Fig. 4:**
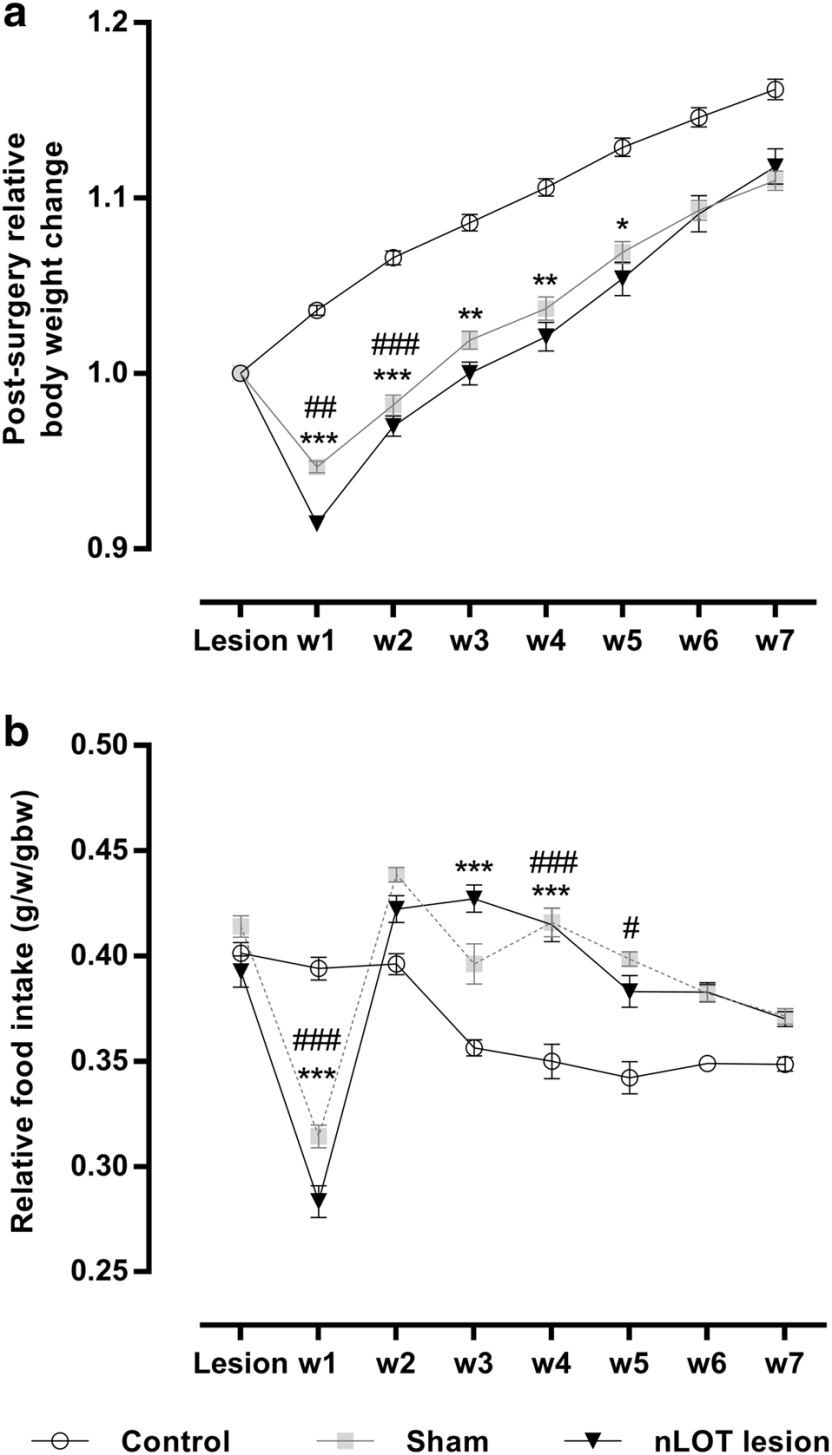
Relative body weight and food intake variations following the day of surgery for inducing nLOT or sham lesions, presented as mean ± SEM. **a** Body weight change, expressed as a percent of the average weekly gain or loss from pre-surgery weight. One week after surgery, nLOT- and sham-lesioned rats showed significant weight loss compared to controls, followed by a progressive increase until the end of the experiments. During week-2 post-surgery, the relative body weight gain was significantly higher in nLOT- and sham-lesioned rats than in controls. From weeks 3 to 5, only in nLOT-lesioned rats the relative weight gain was significantly higher than in controls. Thereafter, the relative body weight variations were similar in all groups. **b** Relative food intake, expressed as grams of food intake per week, per gram body weight. The relative food intake was smaller in nLOT- and sham-lesioned rats compared to controls during week-1 post-surgery. Thereafter, there was a tendency for the relative food intake to be higher in nLOT- and sham-lesioned rats than in controls. However, the differences reached statistical significant levels only at weeks 3 and 4 for nLOT-lesioned rats, and weeks 4 and 5 for sham-lesioned rats. *p < 0.05, **p < 0.01, ***p < 0.001 between nLOT-lesioned and controls; ^#^ *p* < 0.05, ^##^ *p* < 0.01, ^###^ *p* < 0.001 between sham-lesioned and control rats

Body weights were significantly influenced by treatment (*F*_2,87_ = 7.7, *p* < 0.001) and post-surgery time (*F*_1,87_ = 907.0, *p* < 0.001); a significant treatment × post-surgery time was also found (*F*_2,87_ = 17.0, *p* < 0.001). At the beginning of the experiments, rats in all groups had similar body weights (Table 1). During the experiments, the body weight variations relative to pre-lesion body weights (Fig. 4a) were significantly influenced by treatment (*F*_2,87_ = 62.5, *p* < 0.001) and post-surgery time (*F*_7,609_ = 742.8, *p* < 0.001); a significant treatment × post-surgery time interaction was also found (*F*_14,609_ = 29.1, *p* < 0.001). nLOT- and sham-lesioned rats initially showed a significant weight loss, which reached, on average, 8 and 5%, respectively, of the preoperative body weight at 1 week after surgery, followed by a progressive increase until the end of the experiments. During weeks 2–5 post-surgery, the relative body weight gain was significantly higher in nLOT-lesioned rats than in controls (Fig. 4a), whereas for sham-lesioned rats the relative body weight was significantly higher only in week 2. Thereafter and until the day of killing, the relative body weight gain did not significantly differ among nLOT-lesioned, sham-lesioned and control rats. Consequently, at the end of the experiments, nLOT- and sham-lesioned rats were significantly less heavy than control rats (Table 1).

**Table 1:** Summary of the effects of nLOT and sham lesions on body weights, fat mass, leptin and testosterone levels

	Control	Sham	nLOT lesion
Body weight (g)			
Day of surgery	375 (4)	361 (4)	361 (6)
End of experiment	435 (4)*	401 (5)*	404 (8)*
Fat mass (% bw)	4.1 (0.1)	2.9 (0.1)^#^	3.2 (0.1)^#^
Leptin (ng/mL)	5.3 (0.09)	4.3 (0.09)^#^	4.4 (0.06)^#^
Testosterone (ng/mL)	2.1 (0.28)	1.9 (0.26)	2.5 (0.23)

Values are expressed as mean (SEM)

* *p* < 0.001 compared with initial body weight of the respective group

^#^ *p* < 0.001 compared with control rats

Fat mass, expressed as a percentage of body weight, was significantly influenced by treatment (*F*_2,87_ = 25.3, *p* < 0.001). It did not differ between nLOT- and sham-lesioned rats, and was, in both groups, significantly smaller than in controls (Table 1).

### Food intake

The relative food intake (g/week/g body weight) was influenced by treatment (*F*_2,87_ = 8.9, *p* < 0.001) and post-surgery time (*F*_7,609_ = 93.2, *p* < 0.001); a significant treatment × post-surgery time interaction (*F*_14,609_ = 44.0, *p* < 0.001) was also found. Baseline pre-surgery relative food intake was similar in all groups at the beginning of the experiments (Fig. 4b). During week-1 post-surgery, the relative food intake became significantly smaller in nLOT- and sham-lesioned rats than in controls. Thereafter, there was a tendency for the relative food intake to be higher in nLOT- and sham-lesioned rats than in controls. However, the differences reached statistical significant levels only at weeks 3 and 4 for nLOT-lesioned rats, and weeks 4 and 5 for sham-lesioned rats.

### Hormone levels

These data are shown in Table 1. Serum leptin levels were significantly influenced by treatment (*F*_2,87_ = 51.5, *p* < 0.001). They were similar in nLOT- and sham-lesioned rats, but were in both groups significantly lower than in controls. Conversely, serum testosterone levels did not differ among all groups (*F*_2,87_ = 1.3, n.s.).

### nLOT-lesioned rats have severe olfactory deficits

**Fig. 5:**
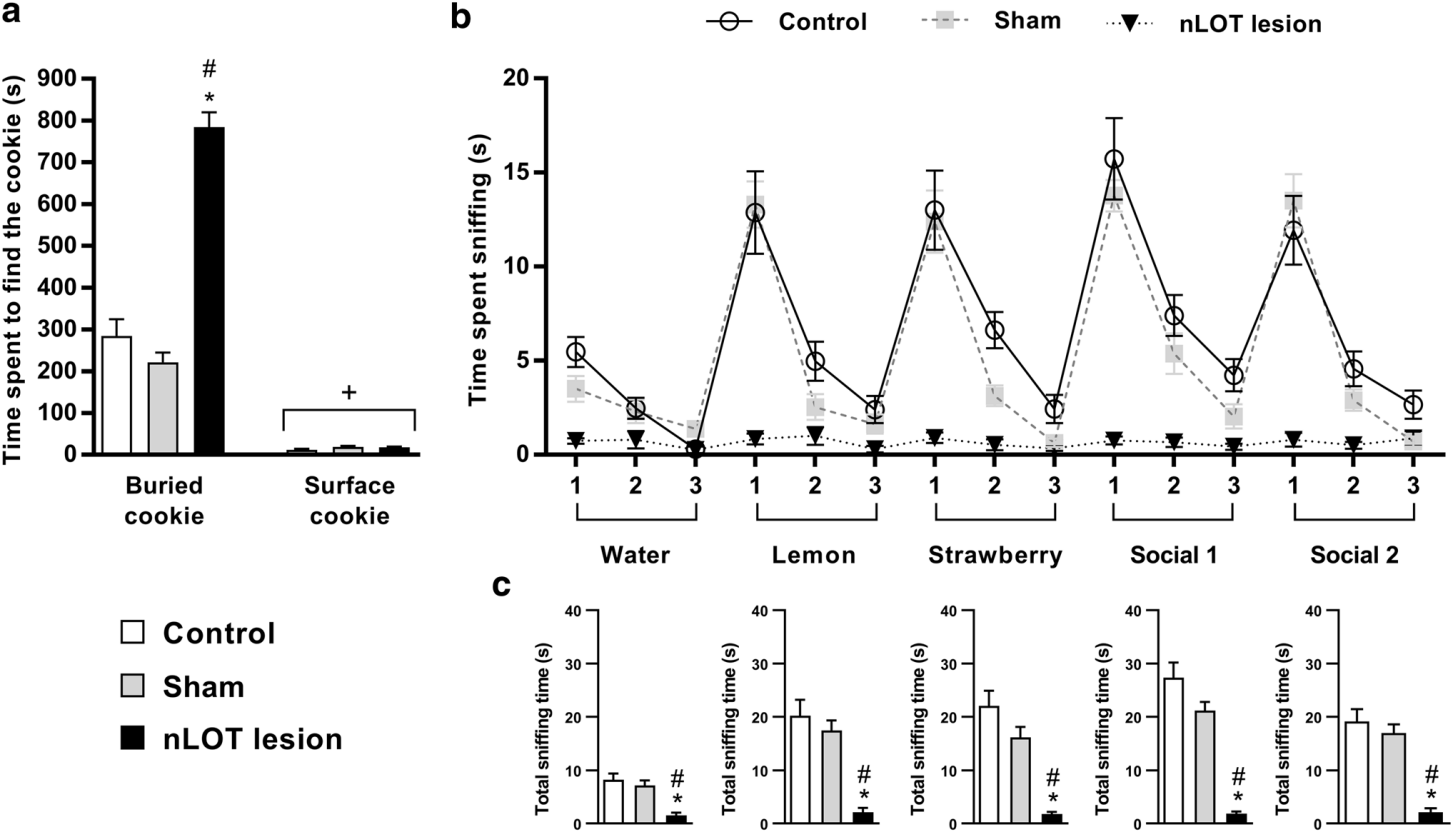
nLOT-lesioned rats have severe olfactory deficits. **a** Buried food test. Histograms show the mean + SEM time spent to find the hidden and the surface cookies. The latency to find the hidden cookie was significantly longer in nLOT-lesioned than in sham-lesioned and control rats. No differences were found between groups to find the visible cookie. **b, c** Olfactory habituation/cross-habituation test. The graph **b** shows mean ± SEM olfactory investigation times across three consecutive 2-min trials separated with 1-min inter-trial of water, two nonsocial and two social odors. Control and sham-lesioned rats, but not nLOT-lesioned rats, show significantly shorter investigation times across three presentations of the same odor (habituation). In addition, control and sham-lesioned rats, but not nLOT-lesioned rats, significantly increased the investigation time after presentation of a new odor (cross-habituation). No significant differences were found between the investigation times of control and sham-lesioned rats. The values of the post-hoc tests are shown in the “Results” section. The histogram **c** shows that the mean + SEM cumulative time that nLOT-lesioned rats spent investigating each odor was significantly inferior to that spent by sham-lesioned and control rats. *p < 0.001 compared to controls; ^#^ *p* < 0.001 compared to sham-lesioned rats; ^+^ *p* < 0.001 compared to the respective group in the buried food test

As shown in Fig. 5a, there was a significant influence of treatment (*F*_2,174_ = 84.3, *p* < 0.001) and cookie location (*F*_1,174_ = 453.9, *p* < 0.001) on the latency to find the cookie; a significant treatment × cookie location interaction (*F*_2,174_ = 83.2, *p* < 0.001) was also found. The average latency to find the hidden cookie was significantly longer in nLOT-lesioned than in sham-lesioned and control rats. No differences were found between sham-lesioned and control rats. Conversely, there were no differences among groups in the time spent to find the visible cookie.

In the olfactory habituation/cross-habituation test (Fig. 5b), repeated measures ANOVA showed that rats in all groups responded to the sequential presentation of the five odors (*F*_14,1218_ = 48.3, *p* < 0.001), and that there was a significant influence of treatment (*F*_2,87_ = 51.0, *p* < 0.001) in the amount of olfactory investigation of the different odors. The analysis of individual odors showed that there was a significant effect of treatment in the response to water (*F*_2,87_ = 15.1, *p* < 0.001), lemon (*F*_2,87_ = 21.4, *p* < 0.001), strawberry (*F*_2,87_ = 26.4, *p* < 0.001), social 1 (*F*_2,87_ = 49.7, *p* < 0.001) and social 2 (*F*_2,87_ = 30.7, *p* < 0.001) odors. Post-hoc analysis of these data revealed that nLOT-lesioned rats spent significantly less time sniffing water, nonsocial and social odors than control and sham-lesioned rats (*p* < 0.001, for all odors) and that there were no differences between control and sham-lesioned rats. As expected, when the cumulative time that animals spent sniffing each odor over the three trials was analyzed, a significant effect of treatment was also found: water (*F*_2,87_ = 15.1, *p* < 0.001), lemon (*F*_2,87_ = 21.4, *p* < 0.001), strawberry (*F*_2,87_ = 26.4, *p* < 0.001), social 1 (*F*_2,87_ = 49.7, *p* < 0.001) and social 2 (*F*_2,87_ = 30.7, *p* < 0.001) odors (Fig. 5c).

When each experimental group was individually analyzed, a significant habituation to each odor was observed in control rats, as shown by the progressive decline in the time spent sniffing water (*F*_2,58_ = 25.6, *p* < 0.001), lemon (*F*_2,58_ = 17.5, *p* < 0.001), strawberry (*F*_2,58_ = 18.0, *p* < 0.001), social 1 (*F*_2,58_ = 17.9, *p* < 0.001) and social 2 (*F*_2,58_ = 16.0, *p* < 0.001) odors. The same happened with sham-lesioned rats for water (*F*_2,58_ = 3.5, *p* < 0.05), lemon (*F*_2,58_ = 82.4, *p* < 0.001), strawberry (*F*_2,58_ = 43.3, *p* < 0.001), social 1 (*F*_2,58_ = 52.3, *p* < 0.001) and social 2 (*F*_2,58_ = 63.2, *p* < 0.001) odors. Conversely, in nLOT-lesioned rats no habituation was found for any of the odors tested: water (*F*_2,58_ = 0.8, n.s.), lemon (*F*_2,58_ = 1.9, n.s.), strawberry (*F*_2,58_ = 2.2, n.s.), social 1 (*F*_2,58_ = 0.7, n.s.) and social 2 (*F*_2,58_ = 0.5, n.s.) odors.

When evaluating cross-habituation, we found that there was a significant effect of treatment when a new odor was presented for the first time: water to lemon (*F*_2,87_ = 25.4, *p* < 0.001), lemon to strawberry (*F*_2,87_ = 20.6, *p* < 0.001), strawberry to social 1 (*F*_2,87_ = 30.2, *p* < 0.001) and social 1 to social 2 (*F*_2,87_ = 35.4, *p* < 0.001) odors. Post-hoc analysis showed that nLOT-lesioned rats responded significantly less than control and sham-lesioned rats when lemon (*p* < 0.001), strawberry (*p* < 0.001), social 1 (*p* < 0.001) and social 2 (*p* < 0.001) odors were presented for the first time. No significant differences were found between control and sham-lesioned rats. When each experimental group was individually analyzed, control and sham-lesioned rats showed a significant cross-habituation when presented for the first time to a new odor: water to lemon (*F*_1,29_ = 33.2, *p* < 0.001 and *F*_1,29_ = 89.0, *p* < 0.001, respectively), lemon to strawberry (*F*_1,29_ = 27.7, *p* < 0.001 and *F*_1,29_ = 39.7, *p* < 0.001, respectively), strawberry to social 1 (*F*_1,29_ = 52.7, *p* < 0.001 and *F*_1,29_ = 246.2, *p* < 0.001, respectively), and social 1 to social 2 (*F*_1,29_ = 14.4, *p* < 0.001 and *F*_1,29_ = 45.2, *p* < 0.001, respectively). In contrast to these groups, nLOT-lesioned rats could not discriminate between any of the odors tested: water and lemon (*F*_1,29_ = 3.1, n.s.), lemon and strawberry (*F*_1,29_ = 3.1, n.s.), strawberry and social odor 1 (*F*_1,29_ = 3.2, n.s.), and social 1 and social 2 (*F*_1,29_ = 0.7, n.s.) odors.

### nLOT-lesioned rats have normal locomotor activity

**Fig. 6:**
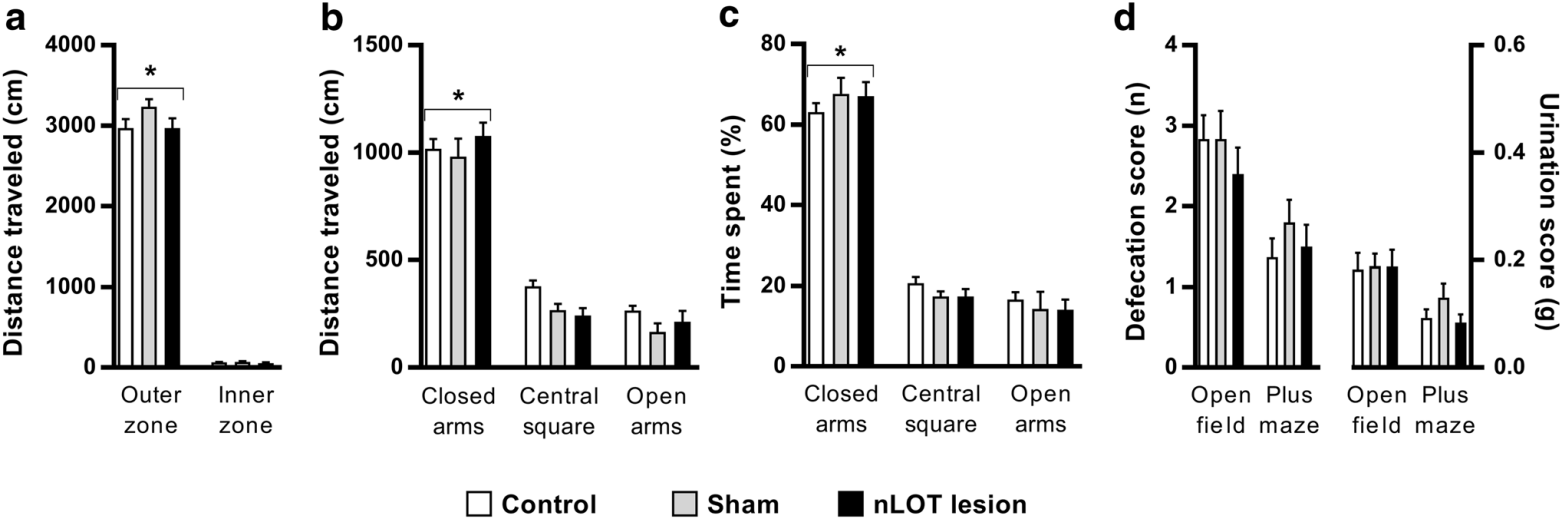
nLOT-lesioned rats have normal locomotor activity and do not exhibit anxiety-like behaviors. **a** Open-field test. The histogram shows the mean + SEM distances travelled in outer and inner zones of the open-field. There were no significant differences between groups in the distances travelled in each zone, and similar to control and sham-lesioned rats, nLOT-lesioned rats travelled significant longer distances in the outer zone than in the inner zone. **b, c** Elevated plus-maze test. Graphic representation of the mean + SEM distances travelled (**b**) and time spent (**c**) in closed and open arms, and in central square of the elevated plus-maze. There were no significant differences between controls, sham-lesioned and nLOT-lesioned rats in the distances travelled and in the time spent in the closed and open arms, and in central square of the maze. In addition, rats of all groups travelled significantly longer distances and spent significantly more time in the closed arms than in the open arms and central square of the maze. **d** The histogram shows the mean + SEM number of fecal boli and amount of urine deposited by rats during the open-field and elevated plus-maze tests. No significant differences between the groups were found. *p < 0.001 compared with the inner zone in the open-field test and with the open arms and central square in the elevated plus-maze test

Locomotor activity was assessed in the open-field and elevated plus-maze (Fig. 6). The distances, expressed in cm (SEM), travelled in the open-field were 3037 (115) for control rats, 3308 (96) for sham-lesioned rats and 3025 (131) for nLOT-lesioned rats. In the elevated plus-maze the total distances were 1652 (52), 1481 (70) and 1534 (61), respectively. There was no significant influence of treatment in the total distances travelled in the open-field (*F*_2,174_ = 2.1, n.s.) as well as in the elevated plus-maze (*F*_2,261_ = 2.2, n.s.), indicating no differences among groups in locomotor and exploratory activities.

### nLOT-lesioned rats do not exhibit anxiety-like behaviors

We measured anxiety-like behavior in the open field and in the elevated plus-maze. There was no significant influence of treatment (*F*_2,174_ = 2.1, n.s.), but there was a significant main effect of zone (*F*_1,174_ = 2173.6, *p* < 0.001) on the distances travelled in the outer and in the inner zones of the open-field; no treatment × zone interaction was found (*F*_2,174_ = 1.7, n.s.). Similar to control and sham-lesioned rats, nLOT-lesioned rats travelled longer distances in the outer zone than in the inner zone of the open-field (Fig. 6a). The distances travelled and the time spent in the different zones of the elevated plus-maze were also significantly influenced by zone (*F*_2,261_ = 262.7, *p* < 0.001 and *F*_2,261_ = 324.9, *p* < 0.001, respectively), but not by treatment (*F*_2,261_ = 2.2, n.s. and *F*_2,261_ = 0.04, n.s., respectively); no treatment × zone interactions were found (*F*_4,261_ = 1.1, n.s. and *F*_4,261_ = 0.8, n.s., respectively). Rats of all groups travelled significantly longer distances (Fig. 6b) and spent significantly more time (Fig. 6c) in the closed arms than in the central square and opens arms of the elevated plus-maze, where rats of all groups spent approximately the same time and travelled approximately the same distances. No differences between groups were found in the open-field and in the elevated plus-maze, which indicates that nLOT lesions do not affect the state of anxiety.

There was also no significant effect of treatment on the defecation and urination scores (Fig. 6d) in the open-field (*F*_2,87_ = 0.6, n.s. and *F*_2,87_ = 0.01, n.s., respectively) as well as in the elevated plus-maze (*F*_2,87_ = 0.7, n.s. and *F*_2,87_ = 1.5, n.s., respectively).

### nLOT-lesioned rats do not have depressive-like behaviors

**Fig. 7:**
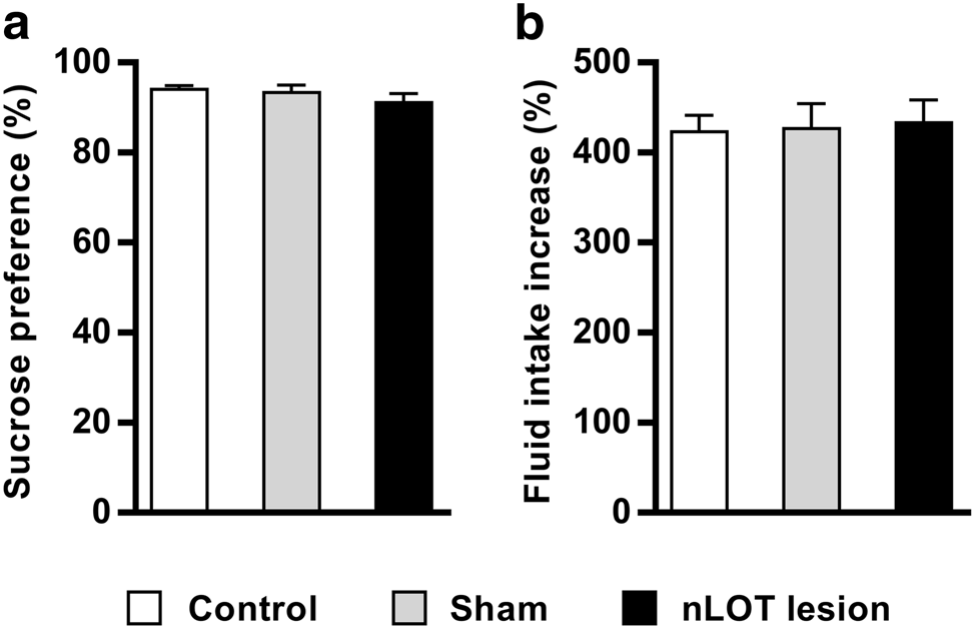
nLOT-lesioned rats do not have depressive-like behaviors. **a, b** Sucrose preference test. The histogram in **a** shows the mean + SEM percentage of sucrose solution ingestion relative to the total amount of liquid consumption by each group of rats averaged over the 4 days of the test. No significant differences were found in sucrose preference between groups, indicating that nLOT lesions do not increase anhedonia or induce depressive-like behaviors. The histogram in **b** shows the mean + SEM percent increase in the total amount of fluid consumption (water plus sucrose solution) during the test relative to baseline water consumption. No differences among all groups were found

No significant effect of treatment on sucrose preference was found among all groups (*F*_2,87_ = 1.5, n.s.; Fig. 7a), which indicates that nLOT lesions do not increase anhedonia and depressive-like responses. As shown in Fig. 7b, there were no differences among groups in the increase in fluid intake that occurred during the 4-day sucrose preference test relative to the baseline fluid intake of each group (*F*_2,87_ = 0.1, n.s.).

### nLOT-lesioned rats have unimpaired fear acquisition and memory

**Fig. 8:**
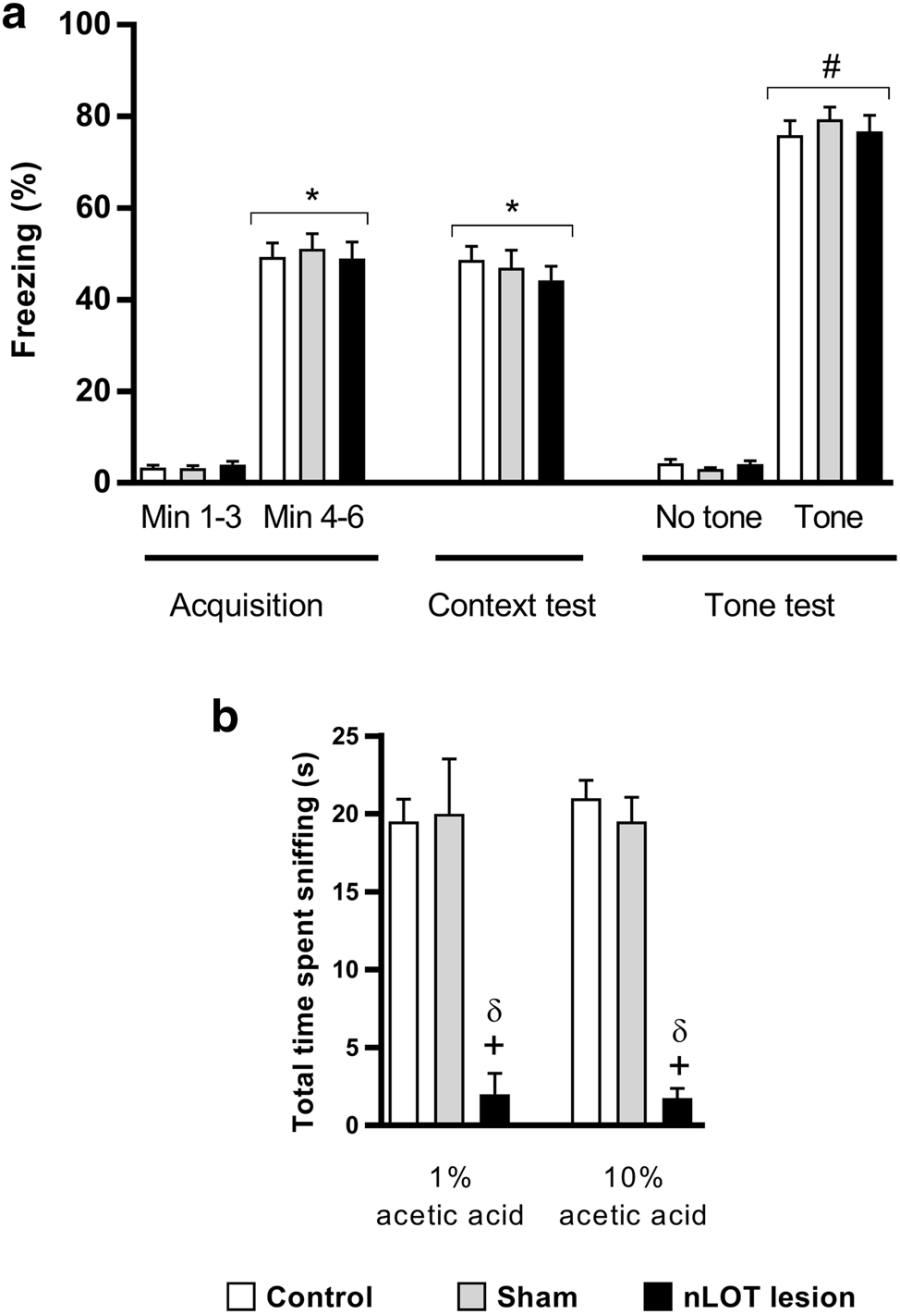
nLOT-lesioned rats have similar levels of contextual and cued fear conditioned learning and memory. **a** nLOT- and sham-lesioned rats did not show any difference compared to control rats in the percentage of freezing time for each of the 3-min periods of the acquisition session, during the context retention test, and during each of the 3-min periods of the tone retention test, which was performed in a novel context. No tone or footshock was delivered during the first 3-min periods of the acquisition session and of the tone retention test. The histogram **b** shows that the mean cumulative time that nLOT-lesioned rats spent investigating 1% and 10% acetic acid was significantly inferior to that spent by sham-lesioned and control rats. Data are presented as the mean + SEM. *p < 0.001 compared to min 1–3 of the acquisition test of the respective group; ^#^ *p* < 0.001 compared to the no tone period of the respective group; ^+^ *p* < 0.001 compared to controls, ^δ^ *p* < 0.001 compared to sham-lesioned rats

Data of the fear conditioning test (Fig. 8a) showed that conditioning increased the total time of freezing in all groups (*F*_1,87_ = 588.6, *p* < 0.001). However, no effect of treatment (*F*_2,87_ = 0.1, n.s.) and no treatment × conditioning interaction (*F*_2,87_ = 0.2, n.s.) was found, which indicates a similar acquisition of fear during training in all groups. Measurement of conditioned fear 24 h post-training upon re-exposure to context revealed that all groups increased the total time of freezing (*F*_1,87_ = 476.6, *p* < 0.001) and that there was no effect of treatment (*F*_2,87_ = 0.4, n.s.) in contextual fear memory, which indicates that all groups have similar levels of contextual memory. When the rats were introduced into the novel context, there was no increase in the total time of freezing in all groups (*F*_1,87_ = 0.3, n.s.). However, when rats were exposed to the conditioned stimulus (tone) there was a significant increase in total freezing time (*F*_1,87_ = 1500.2, *p* < 0.001), and this effect was not dependent on treatment (*F*_2,87_ = 0.2, n.s.) or on treatment × stimulus interaction (*F*_2,87_ = 0.6, n.s.), showing that rats in all groups have similar levels of cued fear memory. To examine the relevance of the acetic acid odor for the normal response of nLOT-lesioned rats in the contextual fear conditioning, we analyzed, using a habituation/cross-habituation test, the ability of nLOT-lesioned rats to smell acetic acid in two different concentrations (1 and 10%). Our results show that there was a significant influence of treatment (*F*_2,18_ = 63.3, *p* < 0.001), but not of acetic acid concentration (*F*_1,18_ = 0.03, n.s.), in the amount of olfactory investigation (Fig. 8b).

### nLOT-lesioned rats do not show innate attractive or avoidance behaviors

**Fig. 9:**
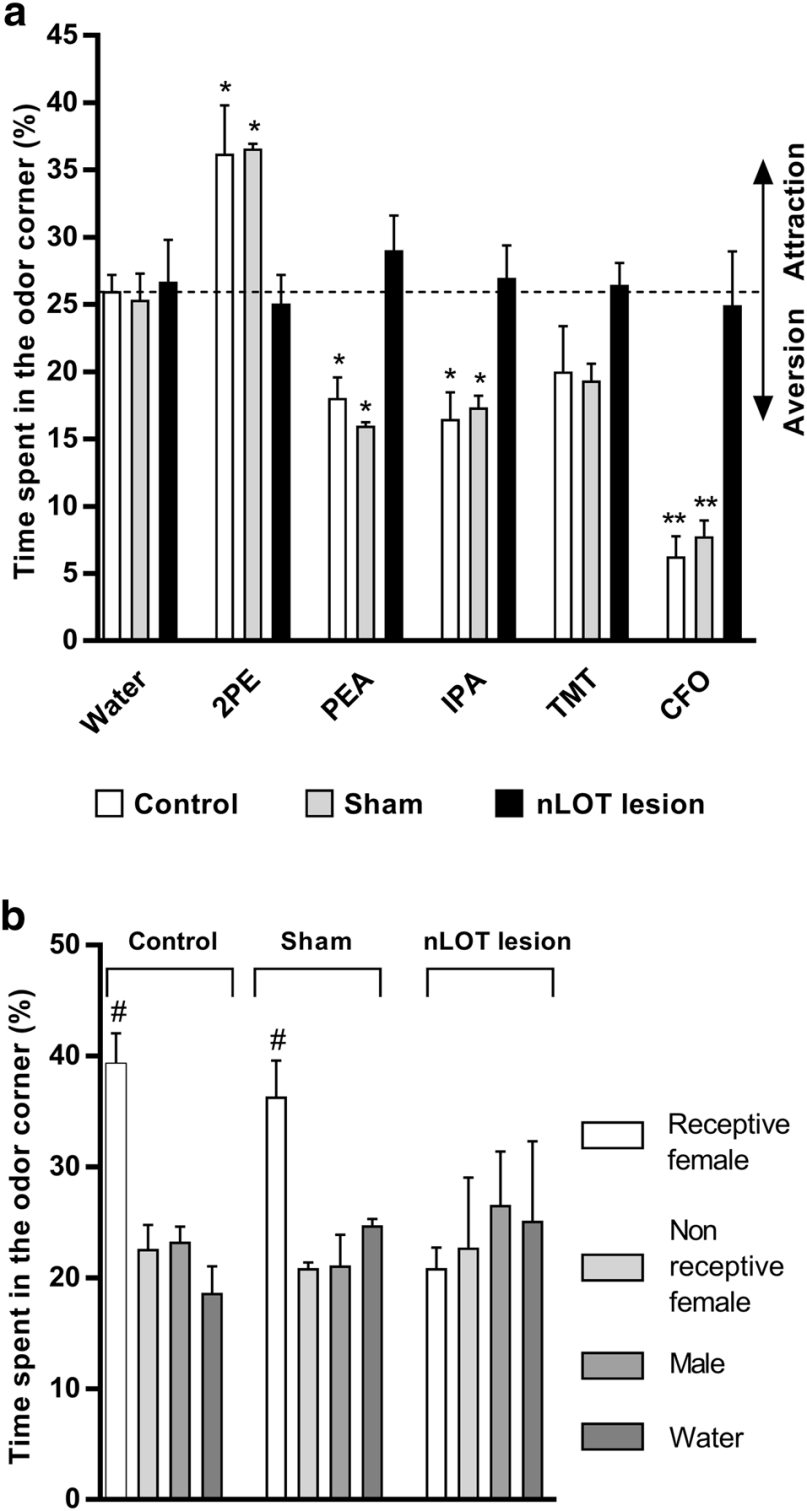
nLOT-lesioned rats do not show innate odor attractive or aversive behaviors. The histogram in **a** shows the mean + SEM percentage of the cumulative time that rats spent investigating the corner of the open-field arena where the several odors were individually presented over the duration of the test. The percentage of time spent investigating water (*dashed line*) was used as the criterion to define the threshold between attraction and aversion. Control and sham-lesioned rats spent significantly more time sniffing the attractive odor 2-phenylethanol (2PE) and significantly less time sniffing the aversive odors 2-phenylethylamine (PEA), isopentylamine (IPA) and cat fur odor (CFO) than water. Conversely, nLOT-lesioned rats spent a similar time investigating water and all the other odors presented. **b** Graphic representation of the mean + SEM percentage of the cumulative time that rats spent investigating the corners of the open-field arena where the Petri dishes containing the odors of receptive females, non-receptive females, males and water (one in each corner) over the duration of the test. Control and sham-lesioned rats spent significantly more time sniffing the receptive female odor than the remaining odors. Conversely, nLOT-lesioned rats did spent a similar time investing all odors presented. *p < 0.05, **p < 0.001 compared to water of the respective group; ^#^ *p* < 0.001 compared to other odors and water

As shown in Fig. 9a, treatment significantly influenced the percentage of time spent in the odor corner of the open-field apparatus, compared to water, when rats were exposed to 2PE (*F*_2,54_ = 3.3, *p* < 0.05), PEA (*F*_2,54_ = 7.4, *p* < 0.01), IPA (*F*_2,54_ = 4.8, *p* < 0.05) and CFO (*F*_2,54_ = 10.4, *p* < 0.001), but not to TMT (*F*_2,54_ = 2.1, n.s.). Similarly, odors also significantly influenced the percentage of time spent in the odor corner, compared to water, when rats were exposed to 2PE (*F*_1,54_ = 12.1, *p* < 0.001), PEA (*F*_1,54_ = 9.4, *p* < 0.01), IPA (*F*_1,54_ = 13.4, *p* < 0.001), TMT (*F*_1,54_ = 4.9, *p* < 0.05) and CFO (*F*_1,54_ = 44.1, *p* < 0.001). Moreover, a significant treatment × odor interaction was also found for 2PE (*F*_2,54_ = 4.7, *p* < 0.05), PEA (*F*_2,54_ = 5.1, *p* < 0.01), IPA (*F*_2,54_ = 3.7, *p* < 0.05) and CFO (*F*_2,54_ = 8.4, *p* < 0.001), but not for TMT (*F*_2,54_ = 1.1, n.s.). Because the behavioral response to TMT is concentration-dependent (Saraiva et al. 2016), we repeated the test with a concentration that was five times higher. The results obtained were similar to those elicited by the concentration of 80 mM (data not shown). No concentration-related effects were also detected for 2PE, PEA and IPA (data not shown). Control and sham-lesioned rats spent significantly more time in the corner containing 2PE and significantly less time in the corner containing PEA, IPA or CFO than in the corner containing water, which indicates that they displayed innate attraction to 2PE and innate aversion to PEA, IPA and CFO. Conversely, in nLOT-lesioned rats, 2PE, PEA, IPA, TMT and CFO did not elicit attractive or avoidance behaviors.

In the triple odor test, we found that the time spent by rats actively investigating the different corners of the open-field arena (Fig. 9b) was significantly influenced by odors in the control (*F*_3,36_ = 17.5, *p* < 0.001) and sham-lesioned (*F*_3,36_ = 11.3, *p* < 0.001) groups, but not in the nLOT-lesioned group (*F*_3,36_ = 0.2, n.s.). Control and sham-lesioned rats showed preference to investigate proestrus female odors over diestrus female or male odors and water, whereas nLOT-lesioned investigated all odors and water for a similar amount of time.

### nLOT-lesioned rats have impaired sexual behavior

**Fig. 10:**
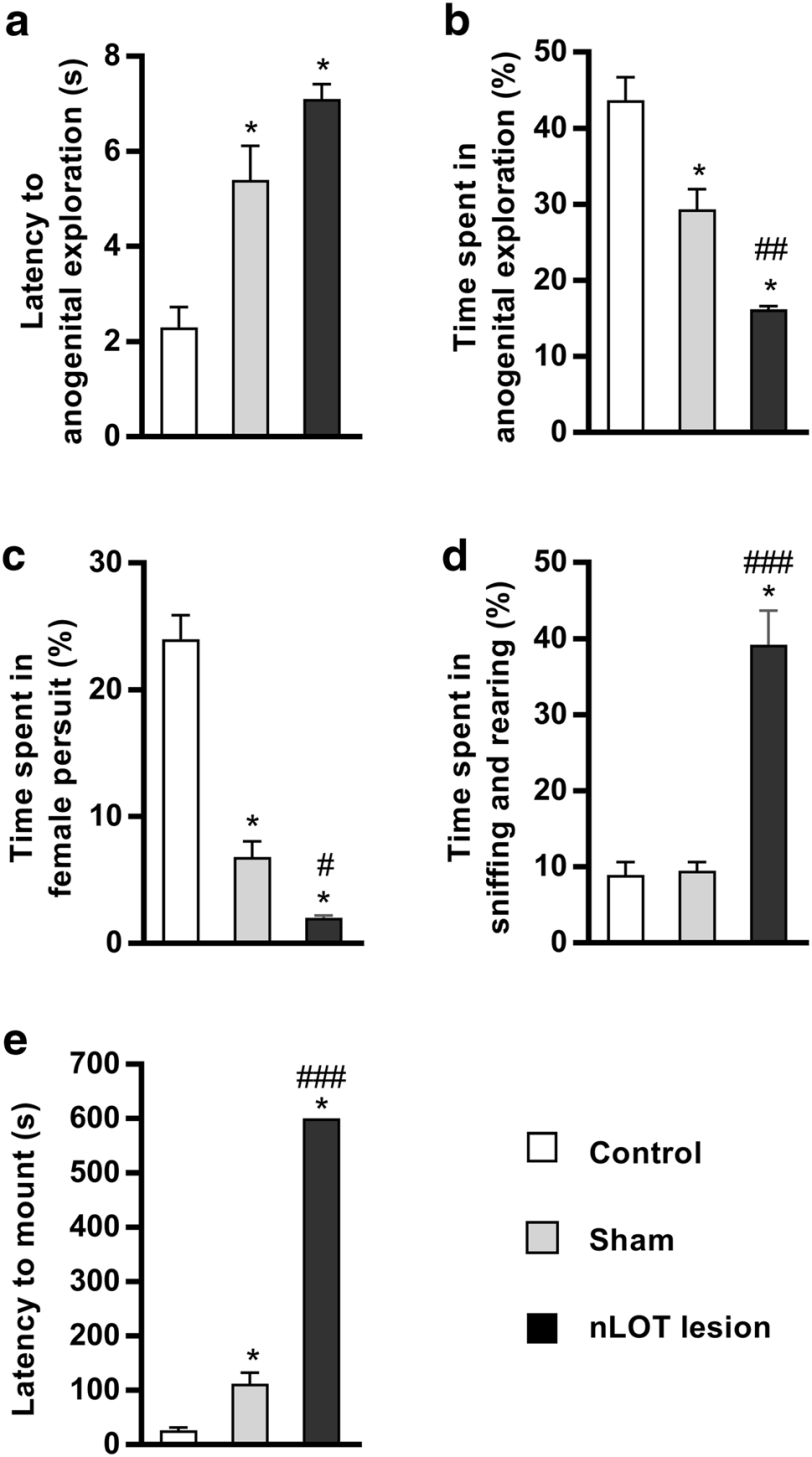
nLOT-lesioned rats have impaired sexual behavior. Histograms show means + SEM values. **a** The latency to the first anogenital exploration of receptive females was significantly longer in nLOT- and sham-lesioned rats than in control. No differences were found between nLOT- and sham-lesioned rats. **b** The percentage of cumulative time over the duration of the test that nLOT-lesioned rats engaged in anogenital exploration was significantly shorter than in sham-lesioned and control rats. Sham-lesioned rats also spent significantly less time in anogenital exploration than controls. **c** The percentage of cumulative time over the duration of the test that nLOT-lesioned rats spent in female pursuit was significantly shorter than that spent by sham-lesioned and control rats. Sham-lesioned rats also spent significantly less time in female pursuit than controls. **d** The percentage of cumulative time over the duration of the test that nLOT-lesioned rats spent in sniffing and rearing behaviors was significantly shorter than that spent by sham-lesioned and control rats. No differences were found in these behaviors between sham-lesioned and control rats. **e** None of the nLOT-lesioned rats exhibited mounting over the 10 min of testing. Contrariwise, all sham-lesioned and control rats exhibited mounting, but the latency to mount was significantly longer in sham-lesioned than in control rats. *p < 0.001 compared to control rats; ^#^ *p* < 0.05, ^##^ *p* < 0.01 and ^###^ *p* < 0.001 compared to sham-lesioned rats

We found a significant influence of treatment in the latency to anogenital exploration (*F*_2,27_ = 22.4, *p* < 0.001) and to the first mount (*F*_2,27_ = 651.6, *p* < 0.001). A similar effect was also found in the percentage of cumulative time spent in anogenital exploration (*F*_2,27_ = 34.5, *p* < 0.001), sniffing and rearing (*F*_2,27_ = 37.9, *p* < 0.001) and female pursuit (*F*_2,27_ = 80.5, *p* < 0.001). The latency to anogenital exploration did not significantly differ between nLOT- and sham-lesioned rats and was, in both groups, significantly longer than in controls (Fig. 10a). The percent time spent by nLOT-lesioned rats in anogenital exploration (Fig. 10b) and in female pursuit (Fig. 10c) was significantly smaller than in sham-lesioned and control rats; in addition, sham-lesioned rats also spent less time in anogenital exploration and in female pursuit than controls. Conversely, the percent time spent in sniffing and rearing did not differ between sham-lesioned and control rats, but was in both groups significantly shorter than in nLOT-lesioned rats (Fig. 10d). All control and sham-lesioned rats exhibited mounting, but the latency to mount was about four times longer in sham-lesioned than in control rats. In contrast, none of the nLOT-lesioned rats exhibited mounting over the 10 min of testing (Fig. 10e).

### nLOT-lesioned rats do not exhibit male–male aggressive behavior

**Fig. 11:**
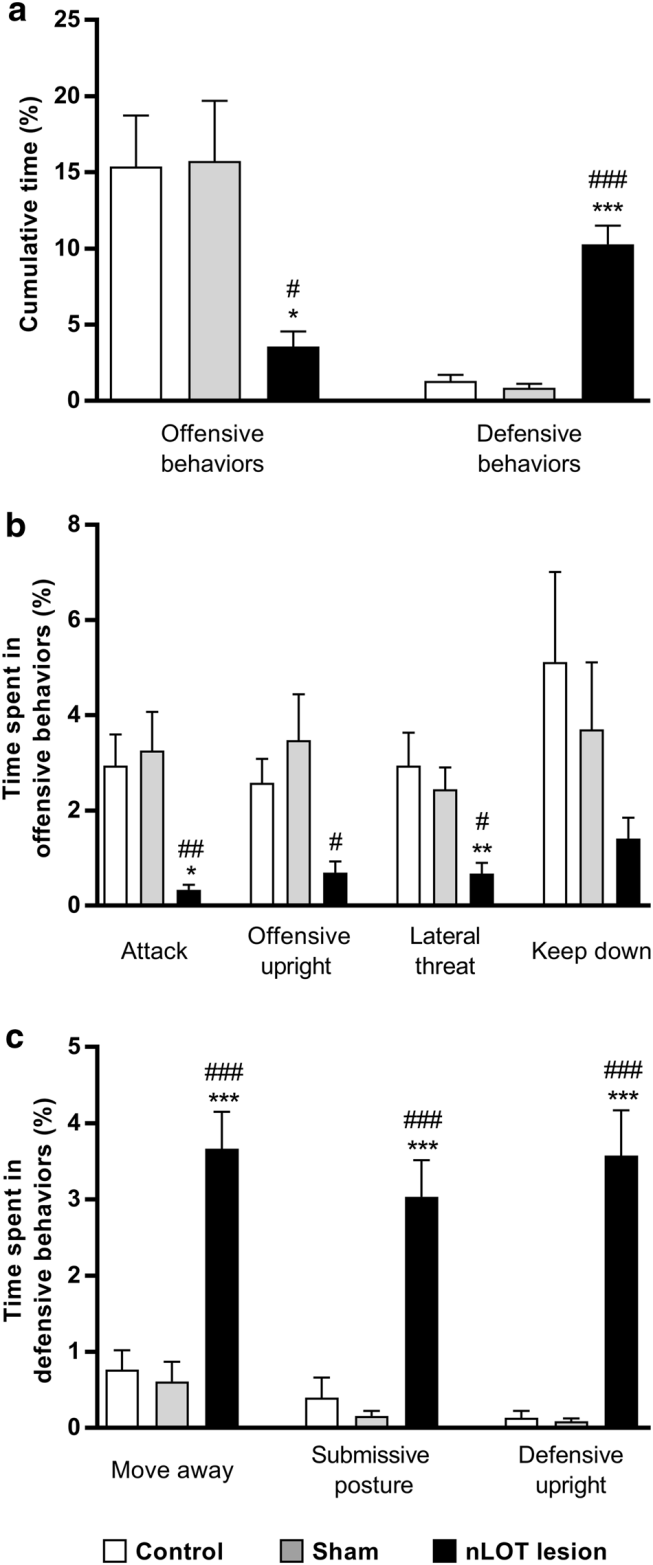
nLOT-lesioned rats do not show aggressive behavior as revealed by the resident-intruder test. Histograms represent means + SEM values of the percentage of the cumulative time over the duration of the test that rats engaged in offensive and defensive behaviors. **a** nLOT-lesioned rats spent significantly less time in offensive behaviors and significantly more time in defensive behaviors than control and sham-lesioned rats. **b** nLOT-lesioned rats spent significantly less time in attack, offensive upright and lateral threat offensive behaviors than control and sham-lesioned rats. **c** Percentage of cumulative time that rats spent in moving away, submissive posture and defensive upright behaviors. nLOT-lesioned rats spent significantly more time in moving away, submissive posture and defensive upright behaviors than control and sham-lesioned rats. *p < 0.05, **p < 0.01 and ***p < 0.001 compared to control; ^#^ *p* < 0.05, ^##^ *p* < 0.01 and ^###^ *p* < 0.001 compared to sham-lesioned rats

We found a significant effect of treatment on the percentage of cumulative time spent in offensive (*F*_2,27_ = 5.2, *p* < 0.001) and in defensive (*F*_2,27_ = 48.3, *p* < 0.001) behaviors. Post-hoc analysis showed that nLOT-lesioned rats spent less time in offensive behaviors and more time in defensive behaviors than control and sham-lesioned rats (Fig. 11a). No significant differences were found between sham-lesioned and control rats. In offensive behaviors (Fig. 11b), we found a significant effect of treatment on the percentage of cumulative time spent in attack (*F*_2,27_ = 7.0, *p* < 0.01), offensive upright (*F*_2,27_ = 4.9, *p* < 0.05) and lateral threat (*F*_2,27_ = 5.8, *p* < 0.01), but not in the keep down behavior (*F*_2,27_ = 1.8, n.s.). nLOT-lesioned rats spent less time in attack, offensive upright and lateral threat behaviors than control and sham-lesioned rats. No significant differences were found between sham-lesioned and control rats. When analyzing defensive behaviors (Fig. 11c), we also found a significant effect of treatment on the percentage of cumulative time spent in move away (*F*_2,27_ = 24.6, *p* < 0.001), submissive posture (*F*_2,27_ = 25.0, *p* < 0.001) and defensive upright (*F*_2,27_ = 33.5, *p* < 0.001). nLOT-lesioned rats spent more time in moving away, submissive posture and defensive upright behaviors than control and sham-lesioned rats. Once again, no significant differences were found between sham-lesioned and control rats.

As opposed to control and sham-lesioned rats, nLOT-lesioned rats did not attack and were not aggressive towards intruder males, and were more submissive, suggesting that nLOT lesions reduce male aggressive behavior.

### nLOT-lesioned rats do not display cognitive alterations

**Fig. 12:**
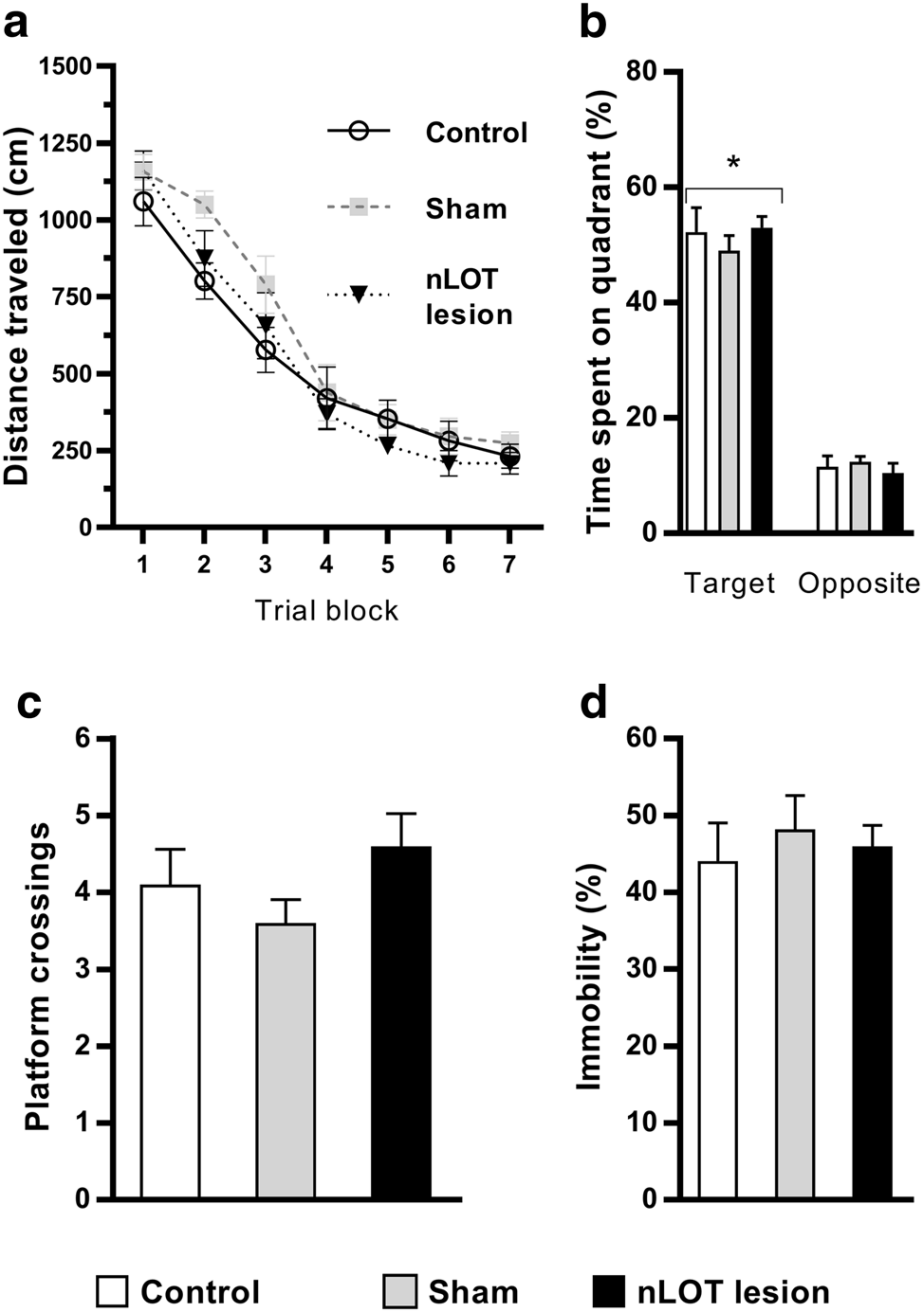
nLOT-lesioned rats do not display cognitive alterations. **a**–**c** Morris water maze test. The graph in **a** shows the mean ± SEM total distances travelled (cm) to find the hidden platform for each block of four consecutive trials in the Morris water maze. There were no significant differences in acquisition performance between groups. The histogram in **b** shows mean + SEM values of the percentage of the cumulative time spent, over the duration of the test, on the quadrant where the hidden platform was located compared to the opposite quadrant. No significant differences were found between the three groups. The histogram in **c** shows mean + SEM number of platform crossings over the duration of the test. No significant differences were found among groups. **d** Forced swim test. The histogram shows the mean + SEM percentage of cumulative immobility time over the duration of the test. No differences were found between nLOT-lesioned, sham-lesioned and control rats. *p < 0.001 compared to the opposite quadrant

The mean distances travelled by rats to find the submersed platform in the reference memory task of the Morris water maze are show in Fig. 12a. Repeated measures ANOVA revealed that rats of all groups progressively improved their ability to locate the hidden platform during the 14 days of acquisition (*F*_6,162_ = 117.0, *p* < 0.001). No differences between the groups were found, as shown by the absence of a significant main effect of treatment (*F*_2,27_ = 1.5, n.s.) and of treatment × trial blocks interaction (*F*_12,162_ = 1.1, n.s.). Behavioral analyses derived from the probe trial are shown in Fig. 12b, c. Two-way ANOVA showed that there was a significant effect of quadrant (*F*_1,54_ = 398.4; *p* < 0.001), but no significant effect of treatment (*F*_2,54_ = 0.1, n.s.) and no treatment × quadrant interaction (*F*_2,54_ = 0.7, n.s.). Rats of all groups spent more time in the target quadrant than in opposite quadrant. Moreover, the time spent in the target quadrant was similar for all groups, revealing that there were no differences among groups on the spatial strategy to search the escape platform during the probe trial. There were also no differences between groups in the number of times that rats crossed the former position of the platform (*F*_2,27_ = 1.5, n.s.; Fig. 12c). Rats of all groups rapidly learned to find the visible platform. The average distances swam over the eight trials to locate the platform position, expressed in cm (SEM), were 225 (58) for controls, 228 (80) for sham-lesioned rats and 206 (66) for nLOT-lesioned rats. No significant differences among the groups were found (*F*_2,27_ = 0.3, n.s.), showing that rats in all groups had similar sensorimotor abilities.

Lesions of the nLOT did not introduce any change in the immobility time evaluated in the forced swim test (*F*_2,27_ = 0.3, n.s.; Fig. 12d), which indicates that lesions of the nLOT do not interfere with the cognitive functions that underlie behavioral adaptation and survival (Molendijk and de Kloet 2015).

## Discussion

The studies reported here were undertaken to determine the extent of functional and behavioral deficits resulting from lesions of the nLOT. Our results show that in male rats, the nLOT plays a role in the sense of smell and in innate behaviors, namely sexual behavior, aggression and predator avoidance. These deficits were observed in the absence of changes in locomotor activity, anxiety, fear, depression and cognitive functions.

nLOT-lesioned rats experienced a weight loss of about 8% during the first week post-surgery. This variation was also apparent in sham-lesioned rats, which indicates that the surgery itself and the stress inherent to it might be responsible for the reduced food ingestion and consequent body weight loss observed in both groups. These changes were transient and followed 1 week later by a progressive increase in food intake and body weights that, however, was insufficient to overcome the weight loss subsequent to surgery. Consequently, at the end of the experiments (i.e., 7 weeks later), control rats were significantly heavier than nLOT- and sham-lesioned rats. The lower body weights of rats in these groups were, in part at least, explained by their smaller amount of adipose tissue. This is consistent with the finding of significantly lower leptin levels in nLOT- and sham-lesioned rats as there is ample evidence that circulating leptin levels tend to parallel adipose tissue mass (Maffei et al. 1995). The nonexistence of differences in all these parameters between nLOT- and sham-lesioned rats indicates that the nLOT might not be especially relevant to the regulation of food intake or energy homeostasis, despite the fact that it expresses the long form of the leptin receptor Ob-Rb (Elmquist et al. 1998), contains melanocortin-expressing neurons (Saito et al. 2001) and projects directly or via multisynaptic pathways to brain regions involved in feeding behavior (Santiago and Shammah-Lagnado 2004).

Particularly impressive in our study was the finding that nLOT-lesioned rats had severe olfactory deficits with inability to detect and discriminate between odors. In the buried food test, nLOT-lesioned rats spent significantly more time to find the hidden cookie than sham-lesioned and control rats. This difference was not due to motor, motivational or other sensory deficiencies because with visible cues present (surface cookie) nLOT-lesioned rats performed the test as well as sham-lesioned and control rats. To better characterize the extent of olfactory impairment in nLOT-lesioned rats, we used the olfactory habituation/cross-habituation test. Control and sham-lesioned rats displayed decreasing durations of investigative behavior when repeatedly exposed to each of the five different odors tested, which is consistent with habituation. Conversely, nLOT-lesioned rats did not display such behavior in any of the odors tested. When a new odor was presented, the duration of investigative behavior was significantly longer in control and sham-lesioned rats, which indicates that they were capable of smelling the new odor (cross-habituation). However, that did not happen with nLOT-lesioned rats, suggesting that they have decreased olfactory sensitivity. The olfactory deficits detected in nLOT-lesioned rats were not consequent to the insertion of the stereotaxic needle as, in both tests, sham-lesioned rats behave similarly to controls. This indicates that the integrity of the nLOT is required for normal olfactory functions.

Odors elicit a variety of innate behaviors that are essential for the survival of the species, namely aversive responses to predator odors (reviewed in Canteras et al. 2015). Because these behaviors are observed in naïve animals, they are believed to be mediated by genetically determined and non-overlapping olfactory circuits (Malnic et al. 1999; Pacifico et al. 2012; Pérez-Gómez et al. 2015). Some nuclei/zones of the olfactory cortical group of the amygdala (Wernecke et al. 2015), and in particular the posterolateral cortical nucleus (Root et al. 2014) that receives spatially stereotyped projections from the main olfactory bulb (Pro-Sistiaga et al. 2007; Sosulski et al. 2011), seem to be essential for mediating approach and avoidance behaviors towards attractive and aversive odors, respectively. Since none of those studies has specifically examined the nLOT, we have addressed this subject herein by analyzing if lesions of the nLOT would interfere with the display of these behaviors, i.e., if nLOT-lesioned rats have, or not, the capacity to differentiate between appetitive and aversive odors. We compared several predator odors whose peripheral detection has been associated with different olfactory structures (main or accessory olfactory systems) or receptors (namely, different trace-amine associated receptors of the main olfactory epithelium, TAARs; Liberles and Buck 2006): PEA, which is present in carnivore urine and specifically activates TAAR4 (Ferrero et al. 2011; Dewan et al. 2013; Liberles 2015); IPA, a biogenic amine produced by leucine decarboxylation that is innately aversive to rodents and activates TAAR3 (Liberles 2015); TMT, a thiazole that is present in fox feces and activates the main olfactory epithelium (Root et al. 2014; Takahashi 2014; Pérez-Gómez et al. 2015); and CFO that primarily activates the vomeronasal organ (Takahashi 2014; Pérez-Gómez et al. 2015). Because, as demonstrated in the posterolateral cortical nucleus of the amygdala, attractive and aversive odors activate different subsets of neurons (Root et al. 2014), and thus the behavioral effects they elicit are likely to be mediated by distinct neural projections, we have also tested the response of nLOT-lesioned rats to the attractive odorant 2PE. Control and sham-lesioned rats demonstrated attraction to 2PE and aversion to all predator odorants, as opposed to nLOT-lesioned rats that failed to show any attractive or avoidance behavior. These experiments demonstrate that nLOT lesions prevent the display of avoidance to predator odors, irrespective of the receptor or olfactory system activated by the odor. Since nLOT neurons do not seem to project to other cortical amygdaloid nuclei known to be involved in avoidance to predator odors, present data suggest that the integrity of the nLOT is required for the expression of this behavior. It must be mentioned here that although TMT induced avoidance behavior in control and sham-lesioned rats, we found no statistical significant differences between these groups when comparing the percentage of time spent investigating TMT and water. This was not related to the concentration of TMT used because the response was similar when the concentration was increased by five times (data not shown). In addition to the characteristics of the synthetic odorant used in this study, the possibility that the rat strain that we have used might have contributed for this finding cannot be ruled out, because as shown in several previous studies (Rosen et al. 2006; Staples and McGregor 2006; Staples et al. 2008; Staples 2010), Wistar rats seem to be less sensitive to TMT than other rat strains. Also, somehow intriguing in our study was the finding that CFO induced a robust avoidance behavior in control and sham-lesioned rats if one considers that the activation of the accessory olfactory system is traditionally thought of as requiring direct contact with the stimulus source. However, the demonstration that exposure to CFO significantly increases the number of FOS-positive cells in the main olfactory bulb (McGregor et al. 2004) raises the possibility that the odor from cat fur might contain volatile sensory cues that can be detected by the olfactory epithelium.

It is nowadays commonly accepted that volatile odors that function as pheromones and influence pheromone-driven behaviors are sensed and processed through both the main and the accessory olfactory systems (Keller et al. 2009; Brennan and Keverne 2015; Brignall and Cloutier 2015; Stowers and Kuo 2015). In rodents, the activation of the main olfactory system by conspecific volatiles seems to be important for social approach and mate recognition (for an overview, see Petrulis 2013; Baum and Cherry 2015). Thus, we next examined if nLOT lesions would interfere with the recognized attraction that sexually inexperienced male rats have to volatile odors from estrus females when compared to diestrus females or males (López et al. 1999; Portillo and Paredes 2004). Our results show that nLOT-lesioned rats have no preference for estrus female odors relative to diestrus female or male odors, indicating that they were not able to detect volatile estradiol-dependent female chemosignals or testosterone-dependent male chemosignals. This behavior contrasts with that observed in control and sham-lesioned rats that spent about twice the time investigating the odor stimulus of estrus over diestrus or male rats. These findings show that lesions of the nLOT interfere with the development of attraction to receptive female odors and, thus, presumably hinder mate recognition. Because volatile chemosignals are primarily sensed by the main olfactory epithelium (for a review, see Petrulis 2013), we can hypothesize that lesions of the nLOT disrupt the neural projections of the main olfactory system that mediate these behaviors.

In addition to receiving inputs from the main olfactory bulb, the nLOT also receives projections from the accessory olfactory bulb (Pro-Sistiaga et al. 2007; Gutiérrez-Castellanos et al. 2014). Since social investigation activates the accessory olfactory system (reviewed in Baum and Cherry 2015; Hashikawa et al. 2016) and this system has an important and complementary role to the main olfactory system (reviewed in Keller et al. 2009; Brennan and Keverne 2015; Brignall and Cloutier 2015; Stowers and Kuo 2015), we have also examined if nLOT-lesioned rats have abnormalities in male-specific behaviors in which the vomeronasal system seems to play an important role, that is, sexual behavior and aggression. Our results show that nLOT-lesioned rats display little interest towards sexually receptive females even when they can interact physically with them, as shown by the increased latency to anogenital exploration, the dramatic reduction in the time spent in anogenital exploration and in female pursuit, and the fourfold increase in the time spent in sniffing and rearing relative to sham-lesioned and control rats. Sham-lesioned rats also spent less time in anogenital exploration and in female pursuit than controls, and their latency to mount was longer than in controls. It is possible that the damage caused by the needle insertion might underlie the behavioral deficits observed in sham-lesioned rats and partially contribute for those displayed by nLOT-lesioned rats. Actually, the trajectory of the needle recurrently included the sublenticular portion of the substantia innominata, a brain region that in addition to being a component of the extended amygdala (McDonald 2003) and projecting to hypothalamic nuclei involved in male sexual behavior (Grove 1988), has been identified as potentially important for regulating social interest in rodents (reviewed in Hashikawa et al. 2016). However, lesions of the nLOT further aggravated the deficits in sociosexual behaviors displayed by sham-lesioned rats, and in sharp contrast with sham-lesioned and control rats, who consistently mounted females, nLOT-lesioned rats failed to display any mounting behavior and, thus, to initiate copulatory behavior.

As expected, in the resident-intruder test, the percent time spent by resident control rats in offensive behaviors towards the intruders was significantly higher than that spent in defensive behaviors. Sham-lesioned rats behaved similar to control rats. Conversely, nLOT-lesioned rats spent approximately three times more time in defensive behaviors towards the intruder than in offensive behaviors. In addition, the time spent by nLOT-lesioned rats in offensive behaviors was just about one quarter of that spent by control and sham-lesioned rats in those behaviors. It is known that sexual and aggressive behaviors in males are influenced by the circulating levels of testosterone (Hull and Dominguez 2007; Albers 2012; Yang and Shah 2014). However, this is not a likely cause of the behavioral changes displayed by nLOT-lesioned rats because their serum concentrations of testosterone did not differ from those of sham-lesioned and control rats. Studies in male mice with complete loss of the main olfactory epithelium functions (Mandiyan et al. 2005; Wang et al. 2006) or chemical ablation of this epithelium (Keller et al. 2006) showed changes in chemoinvestigatory behavior, mounting and aggression similar to those found in nLOT-lesioned rats. Conversely, mutant male mouse strains with only partial defects in the main olfactory system and no general defects in olfaction display deficits in several social behaviors, including chemoinvestigatory preference and aggression, but not in mounting behavior (Matsuo et al. 2015). In light of these data, we can conclude that lesions of the nLOT interfere with the chemosensory processing mediated by the main and the accessory olfactory systems that is required for the display of copulatory and aggressive behaviors in male rats.

Because the behavioral alterations that we have detected in nLOT-lesioned rats might be linked to increased levels of anxiety-like behavior, we have assessed locomotor and exploratory activities in all groups of rats. We found no side effects of nLOT lesions on locomotor activity or general state of anxiety, as demonstrated by the absence of differences between groups in the tests performed in the open-field and elevated plus-maze, namely in the distances travelled in the outer and in the inner zones of the open-field as well as in the open arms, closed arms, and in the central square of the elevated plus-maze or in defecation and urination scores. The behavioral changes displayed by nLOT-lesioned rats were not also attributable to anhedonia because nLOT lesions had no effect on the sucrose preference test. Cognitive abilities evaluated in the Morris water maze were intact, suggesting that the hippocampal-dependent learning and memory are not affected by nLOT lesions. Likewise, nLOT lesions did not interfere with the cognitive functions that underlie stress coping and adaptation, as evaluated by the forced swim test, in which the mesoaccumbens dopaminergic circuit, which is under the control of the hippocampus and amygdala and receives inputs from the nLOT, plays an important role (Molendijk and de Kloet 2015; de Kloet and Molendijk 2016). Also contextual and cued fear conditioned learning and memory, in which one of the projection areas on the nLOT, the basolateral amygdala, plays a critical role (Kim and Jung 2006; Curzon et al. 2009), were unaltered in nLOT-lesioned rats. This was somehow surprising in view of the fact that odor was one of several sensory modalities that defined the context. However, and similar to other odors, nLOT-lesioned rats were unable to smell acetic acid, the odor used in contextual fear conditioning, irrespective of its concentration. Therefore, the normal level of contextual fear in nLOT-lesioned rats suggests that these rats may have increased reliance on visual and tactile cues at the expense of olfactory cues, a possibility that is supported by data from a recent study (Huckleberry et al. 2016) showing that changing only the floor of the test box is sufficient to render two contexts discriminable. The absence of changes in these behaviors shows that the consequences of bilateral lesions of the nLOT differ from those induced by bilateral olfactory bulbectomy because, in this experimental condition, anosmia and reduced sexual activity are associated to hyperactivity, anhedonia and marked cognitive decline (Song and Leonard 2005; Hendriksen et al. 2015).

To conclude, we describe here for the first time functional and behavioral effects of excitotoxic lesions of the nLOT in adult sexually naïve male rats. While some olfactory-related deficits might be expected due to the mixed chemosensory information it receives through both olfactory and vomeronasal projections, the extent and the nature of the effects of nLOT lesions were astounding. They included a complete loss of the sense of smell, with incapacity to identify and discriminate between odors. Possibly due to these effects on general olfactory abilities, nLOT lesions also prevented the display of innate odor-driven behaviors that are critical for species survival and reproduction. We do not know the reasons for the extent and severity of these effects, and we can only speculate at this point that, irrespective of the particular functional attributes of each component of the olfactory system, the normality of olfactory functions and olfactory-driven behaviors seems to require the integrity of all components of the olfactory system.
